# Postbiotics as Molecules Targeting Cellular Events of Aging Brain—The Role in Pathogenesis, Prophylaxis and Treatment of Neurodegenerative Diseases

**DOI:** 10.3390/nu16142244

**Published:** 2024-07-12

**Authors:** Pola Głowacka, Katarzyna Oszajca, Agnieszka Pudlarz, Janusz Szemraj, Monika Witusik-Perkowska

**Affiliations:** 1 Department of Medical Biochemistry, Medical University of Lodz, 6/8 Mazowiecka Str., 92-215 Lodz, Poland; pola.glowacka@umed.lodz.pl (P.G.); katarzyna.oszajca@umed.lodz.pl (K.O.); agnieszka.pudlarz@umed.lodz.pl (A.P.); janusz.szemraj@umed.lodz.pl (J.S.); 2 International Doctoral School, Medical University of Lodz, 90-419 Lodz, Poland

**Keywords:** postbiotics, probiotics, bacteria metabolites, neurodegeneration, Alzheimer’s disease, Parkinson’s disease, bacterial extracellular vesicles, CNS

## Abstract

Aging is the most prominent risk factor for neurodegeneration occurrence. The most common neurodegenerative diseases (NDs), Alzheimer’s (AD) and Parkinson’s (PD) diseases, are characterized by the incidence of proteinopathy, abnormal activation of glial cells, oxidative stress, neuroinflammation, impaired autophagy and cellular senescence excessive for the patient’s age. Moreover, mitochondrial disfunction, epigenetic alterations and neurogenesis inhibition, together with increased blood–brain barrier permeability and gut dysbiosis, have been linked to ND pathogenesis. Since NDs still lack curative treatment, recent research has sought therapeutic options in restoring gut microbiota and supplementing probiotic bacteria-derived metabolites with beneficial action to the host—so called postbiotics. The current review focuses on literature explaining cellular mechanisms involved in ND pathogenesis and research addressing the impact that postbiotics as a whole mixture and particular metabolites, such as short-chain fatty acids (SCFAs), lactate, polyamines, polyphenols, tryptophan metabolites, exopolysaccharides and bacterial extracellular vesicles, have on the ageing-associated processes underlying ND occurrence. The review also discusses the issue of implementing postbiotics into ND prophylaxis and therapy, depicting them as compounds addressing senescence-triggered dysfunctions that are worth translating from bench to pharmaceutical market in response to “silver consumers” demands.

## 1. Introduction

Aging, a phenomenon that cannot be treated as a pathology per se, is considered the most critical risk factor for neurodegenerative diseases (NDs), including Alzheimer’s and Parkinson’s disease, which are the most common neuropathologies in the elderly.

The main hallmark of Alzheimer’s disease (AD) is the accumulation of plaques of amyloid β (Aβ) peptide and neurofibrillary tangle aggregates of hyperphosphorylated, misfolded tau protein, causing an adverse effect on neuronal functions and eventually leading to their degeneration and progressive cognitive impairment [1].

A major clinical manifestation of Parkinson’s disease (PD) is the loss of motor control due to the degeneration of dopamine-producing neurons in the substantia nigra caused by the accumulation of misfolded α-synuclein. However, recent evidence shows that degeneration also affects other regions of the nervous system, explaining the spectrum of sensory, neuropsychiatric, sleep and autonomic abnormalities observed in patients [2].

Apart from proteinopathies afflicting neurons, both NDs are characterized by the presence of abnormally activated microglia and astrocytes, suggesting the involvement of non-neuronal cells in disease pathogenesis and progression [3,4].

Although both diseases are described by different clinical pictures, there are some convergent events that contribute to their neuropathogenesis and ultimately lead to functional degeneration of the CNS. Oxidative stress, neuroinflammation, impaired autophagy, cellular senescence and protein misfolding are processes that accompany brain ageing and have also been implicated as triggers and/or accelerators of the pathology of NDs. Importantly, the complex interplay of these mechanisms, extensively discussed in recent reviews, demonstrates their self-reinforcing power, which is virtually impossible to break with current therapeutic strategies.

However, one of the current research trends is focused on finding therapies with general neuroprotective potential that reduce cellular damage within the CNS and delay disease progression. The use of exogenous antioxidants to modulate oxidative stress may be one strategy for the treatment of ND. Effective antioxidant agents should not only possess the necessary physicochemical properties to cross the BBB but should also have the ability to concentrate in the mitochondria of the diseased tissue or cells, as the mitochondria are largely responsible for generating oxidative stress in neurodegeneration. Scientists have also made efforts to devise successful autophagy-promoting strategies which would cease the proteinopathies spread in the CNS of affected individuals and clear the accumulating misfolded proteins. Another therapeutic target constitutes triggering neurogenesis to replenish damaged neurons in ND patients.

**Figure 1:**
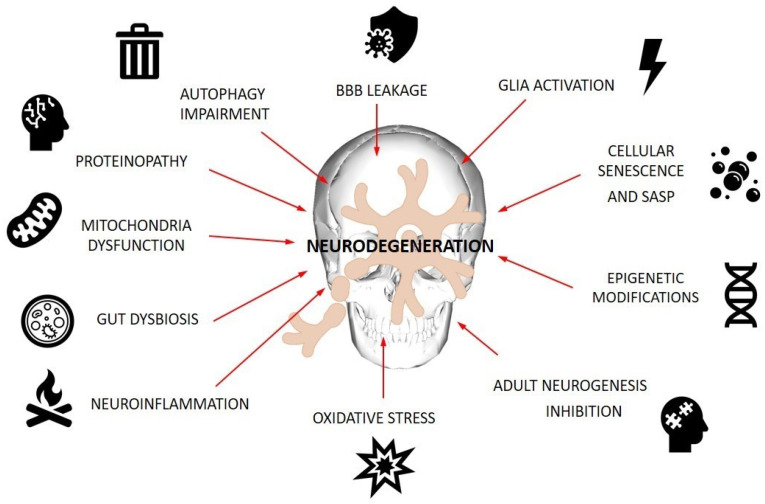
Phenomena involved in neuropathogenesis. The intricate and interrelated mechanisms of neuropathogenesis are not yet fully elucidated and have been observed to aggravate one another, creating vicious circles further expediting occurring pathology. Often, seemingly infinitesimal changes and injuries to the CNS cells accumulate over years, driving the pathogenesis progression, only to be finally diagnosed when the patient’s condition is dire and there is no hope of decelerating the developing disease.

Regarding complex nature of ND pathogenesis, a successful, well-tailored therapy should address multiple concerns at once, for dealing with a sole issue would not be enough to stop the disease progress and amend the neural damage underlying the cognitive deficits suffered by ND patients. The most prominent and discussed in current literature processes contributing to neurodegeneration progression are presented in Figure 1.

Numerous compounds of natural origin exhibiting cytoprotective activity based on their antioxidant, anti-inflammatory and anti-senescent properties appear to be promising candidates for the support of CNS function. Over the recent years, there has been a particular focus on probiotic bacteria and their metabolic by-products, which have been demonstrated to exert a broad range of pro-health effects, including a positive influence on the mental and cognitive abilities of the aging brain. A number of recently published reviews provide an overview of the original contributions to the field, demonstrating the positive impact of probiotics and their metabolomes on CNS functions in health and diseases, including neurodegenerative states. The most widely researched and discussed topic is the impact of the intestinal microbiome on the gut–brain axis, with a particular focus on the potential benefits of probiotic supplementation. However, the latest publications are increasingly turning their attention to microbial metabolites as potential agents directly responsible for a range of beneficial effects [5,6,7,8,9]. This scientific trend has also been translated to the pharmaceutical industry, which offers commercial products named “psychobiotics” and “gerobiotics”. The growing scientific interest in probiotic by-products has quickly yielded commercially available supplements on the market, which are composed of postbiotics only, including compounds with a proven positive influence on neuropsychiatric functions, such as short-chain fatty acids (SCFAs). As the scientific background of this field rapidly expands, the objective of our review is to synthesize the neurodegenerative processes underlying the pathologies of AD and PD in the context of the brain aging phenomenon. Such an approach is to demonstrate these events as potential targets for postbiotics, encompassing not only the most well-known interplay with the metabolic pathways of the host but also newer, preliminarily explored interactions. We emphasized the interrelation of cellular events and its self-reinforced role in the destroying machinery of ND and highlighted the significance of epigenetic mechanisms influencing brain aging. A comprehensive review of postbiotic properties, addressed to a particular cellular piece of the neuroinflammaging puzzle promoting ND, clearly exposes their promising neuroprotective potential. On the other hand, the extensive research in this field still opens the new, unexplored area of the relationship between microbes and their hosts, touching on less explored bacterial metabolites, such as bacterial extracellular vesicles and inhibitors of quorum-sensing peptides, as well as delving into current investigations regarding recently uncovered epigenetic-regulating properties of already thoroughly examined compounds such as butyrate and lactic acid.

## 2. Cellular Events Involved in Pathogenesis of ND

### 2.1. Oxidative Stress and Neuroinflammation

Oxidative stress (OS), resulting from an imbalance between excessive generation of free radicals and an inadequate antioxidant defense system, is considered a key modulator of neurodegenerative diseases. The proper functioning of the central nervous system (CNS) is entirely dependent on the chemical integrity of the brain. High concentrations of and chronic exposure to reactive oxygen species (ROS) cause damage to cellular macromolecules such as DNA, lipids and proteins, ultimately leading to necrosis and apoptotic neural cell death. There are several reasons for the increased sensitivity of neuronal tissue to oxidative stress [10]. Primarily, the metabolically active central nervous system uses large amounts of oxygen to produce ATP in the mitochondrial respiratory chain, which is associated with free radical leakage. Moreover, the brain is enriched in redox-active metals (copper, iron, zinc and manganese) that actively participate in ROS generation by the Fenton reaction [10,11]. In addition, neuronal membranes, rich in polyunsaturated fatty acids, are highly susceptible to oxidative stress due to lipid peroxidation [12]. Nucleic acids are another group of molecules that are biological targets for free radicals. The resulting DNA breaks or modified bases can lead to abnormal gene expression and cell death. Oxidative modification of proteins, in turn, can disrupt the function of enzymes, receptors, neurotransmitters and structural proteins.

Oxidants with harmful effects on neurons include hydrogen peroxide (H_2_O_2_), superoxide anions (O_2_^−^) and highly reactive hydroxyl radicals (HO^•^) as well as nitric oxide (NO), which belongs to the reactive nitrogen species (RNS) [12]. Under normal conditions, endogenous antioxidants are involved in the scavenging of these reactive species to ensure normal cell function. The cellular antioxidant system, designed to protect against tissue damage, consists of enzymes and other non-enzymatic compounds with reducing properties, which are responsible for maintaining oxidation–reduction homeostasis and mitigating OS. The brain has a relatively low antioxidant capacity compared to other tissues, which further increases its vulnerability to oxidative stress [10]. Insufficient antioxidant defense can also lead to brain cell damage.

There is a wealth of scientific evidence that shows a significant connection between OS and neurodegenerative diseases such as Alzheimer’s disease (AD) and Parkinson’s disease (PD) [13,14,15,16,17].

In Alzheimer’s disease, accumulation of reactive species promotes redox imbalance and Aβ- or tau-induced neurotoxicity. In general, oxidative stress-induced Aβ formation and aggregation facilitates the phosphorylation and polymerization of tau and thus leads to the initiation and progression of AD [18,19]. Furthermore, high levels of OS disturb the proper functioning of astrocytes responsible for maintaining the blood–brain barrier (BBB) and removing amyloid plaques, leading to an impaired BBB and Aβ neutralization. In addition, increased levels of reactive species stimulate the transcription of pro-inflammatory genes and the release of cytokines (e.g., IL-1, IL-6, TNF-α) and chemokines, resulting in the development of neuroinflammatory processes. In turn, inflammatory reactions further activate microglia and astrocytes to produce high amounts of ROS, which is the main cause of chronic OS [20].

In Parkinson’s disease, dopaminergic neuronal degeneration is contributed to by oxidative damage and mitochondrial dysfunction. ROS harm the substantia nigra through the oxidation of lipids, proteins and DNA. This effect appears to be triggered mainly by changes in brain iron (Fe) and copper (Cu) content, dopamine (DA) metabolism, mitochondrial dysfunction, activation of monoamine oxidase (MAO) and by changes in the antioxidant defense system [20]. In the presence of an excess of copper and iron ions, brain dopamine undergoes auto-oxidation to form superoxide, hydrogen peroxide, and o-quinones, which are reactive intermediates for the generation of more damaging compounds, including 6-hydroxydopamine (6-OHDA) and R-salsolinol. Both substances are known to be neurotoxins that enhance oxidative stress and mitochondrial damage by inhibiting electron transport chain (ETC) function, which may consequently induce apoptosis of dopaminergic neurons [21]. In addition, the pathogenesis of PD may involve redox pathways such as androgen receptor-induced neurodegeneration, increased aggregation of α-synuclein and production of its oxidatively modified forms, degradation of quinone oxidoreductase 1 (NQO1), reduced activity of deglycase DJ-1, activation of the leucine-rich repeat kinase 2 (LRRK2) gene and impairment of tetrahydrobiopterin and tyrosine hydroxylase (TH) metabolism [21].

A contributing factor that is inextricably linked to oxidative stress in the pathogenesis and progression of neurodegenerative disorders is neuroinflammation. Inflammatory cells secrete ROS and RNS that can further promote intracellular signaling cascades, resulting in an increased expression of pro-inflammatory genes [22]. Neuroinflammation can be characterized as the inflammatory response of the CNS to agents that act against homeostasis. This response involves different cell types, such as astrocytes and microglia. Both can be classified into two opposing phenotypes: neurotoxic and neuroprotective. Alterations in the phenotype of microglia and astrocytes, their loss of neuroprotective function and the development of neurotoxic functions are complex and may vary according to the stage and severity of the ND [23]. The main feature of neuroinflammation is the activation of microglia that results from an imbalance in brain homeostasis occurring due to various pathological conditions (e.g., toxic compounds, injurious and pathogens). Activation of microglia triggers the release of several inflammatory and cytotoxic compounds responsible for neuroinflammation and consequent neurodegeneration. However, microglia can also exhibit neuroprotective effects. For example, in AD, they remove Aβ deposits by phagocytosis [24]. When microglia cease to exert their beneficial effect, inflammation develops, synapses are lost and neurons are damaged. Pro-inflammatory cytokines released from pathogens or damaged cells activate the resting microglia to express pro-inflammatory factors such as IL-1β, IL-6, TNF-α, nitric oxide (NO) and proteases, which have deleterious effects in ND. In turn, IL-4, IL-10, IL-13 and TGF-β activate the neuroprotective properties of microglia, leading to the release of various microglial factors associated with neuroprotection and tissue healing (e.g., IGF-1 or arginase 1).

In the case of Alzheimer’s disease, dysfunctions of microglial and astrocytic metabolism can result in the accumulation of Aβ. Aβ in turn activates microglia and astrocytes to release neuroinflammatory mediators that promote neurodegeneration. Pro-inflammatory cytokines reduce the phagocytic activity of microglia and possibly convert microglia to a pro-inflammatory phenotype. Moreover, pro-inflammatory microglia increase tau phosphorylation and enhance tau pathology [23]. In turn, reactive pro-inflammatory astrocyte phenotypes over-release neurotoxic agents and are associated with synaptic degeneration and glutamate dysregulation. These phenotypes sense Aβ aggregates, which leads to the activation of downstream target genes and subsequent increased production of harmful neurotoxic factors, including ROS, NO and cytokines, such as IL-1β, IL-6, TNF-α or IFN-α [23]. Activation of glial cells may link the initial aggregation of Aβ to the subsequent development of tau aggregates, as microglia activation precedes tau aggregation and supports the hyperphosphorylation of the tau protein, which then leads to the generation of neurofibrillary tangles (NFTs). The tau tangle accumulation in the nervous fibers results in the loss of neuronal function and, finally, apoptosis and activation of immune cells [25]. However, pathogenic protein aggregation and neuroinflammation can mutually promote each other to accelerate the damage inflicted on brain tissue. It is still not clear which one is the initiator of neurodegeneration.

Inflammatory processes also play a key role and may be an early event in Parkinson’s disease. Postmortem studies of PD patients have revealed an increased proportion of active microglia in the substantia nigra. Excessive and irregular activation of microglia results in the release of pro-inflammatory cytokines (including TNF-α, IL-1β and IFN-γ) and ROS, as well as apoptosis activation. Since DA neurons express a wide range of cytokine and chemokine receptors, it has been suggested that they respond to these mediators, which contributes to their degeneration [26,27]. During the disease progression, molecules released from degenerated DA neurons, such as α-synuclein, ATP and metalloproteinase-3 (MMP-3), further increase microglia activation, exacerbate the neuroinflammatory responses in the brain and aggravate neurodegenerative processes, creating a vicious cycle of neurodegeneration. Moreover, overexpression of α-syn causes the toxic accumulation of α-syn fibrils, a major component of Lewy bodies and neurites, which are toxic and trigger the loss of dopaminergic neurons in PD [28]. Furthermore, pro-inflammatory cytokines released from activated microglia can induce phosphorylation of α-synuclein at Ser129, accelerating its fibrillation and the formation of insoluble aggregates [29,30].

Many studies report that astrocytes also play an important role in the neuroinflammatory processes of PD. Like microglia, astrocytes respond to inflammatory triggers, such as LPS, IL-1β and TNF-α, by releasing pro-inflammatory cytokines. Reactive astrogliosis, hallmarked by increased levels of glial fibrillary acidic protein (GFAP) expression, has been noted in PD patients [31]. Astrocytes are activated by various molecules, including pro-inflammatory mediators released from activated microglia, and these signals are then further amplified by astrocytes. The synergistic activation of microglia and astrocytes leads to uncontrollable neuroinflammation and contributes to the increased death of DA neurons in the substantia nigra during neurodegeneration [28].

### 2.2. Autophagy

**Figure 2:**
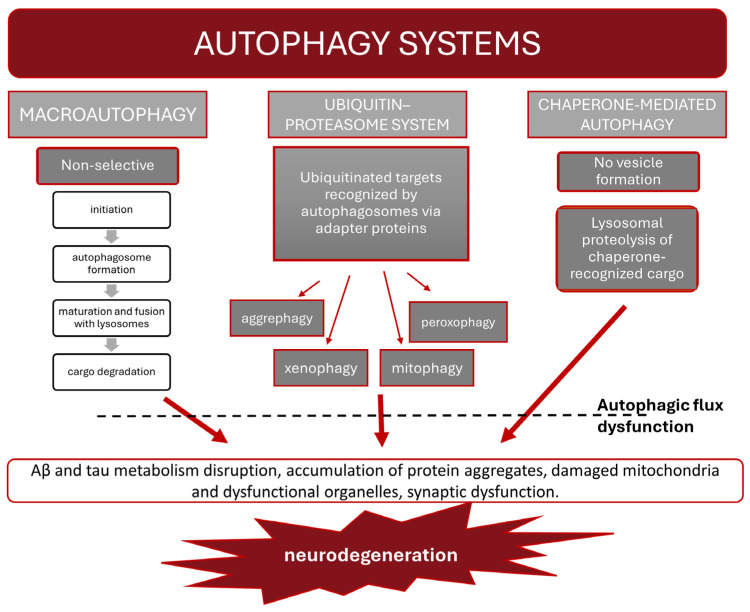
The block of various autophagy systems in ND pathogenesis. Distinguished autophagy systems based on different cellular machinery which are involved in homeostasis maintenance under various external and internal insults and crucial to CNS proper functioning. The dysfunction of autophagic flux, resulting in autophagy impairment, occurs in ND and was observed to contribute to neuropathology via several interrelated mechanisms.

To maintain homeostasis and survive under starvation, oxidative stress and inflammation, conditions created by misfolded proteins or damaged organelles, cells can activate an autophagy program. So far, three systems of autophagy have been identified, which are distinguished by the involvement of different cellular machinery: macroautophagy, the ubiquitin–proteasome system (UPS) and chaperone-mediated autophagy (CMA). The autophagy systems’ division is depicted in Figure 2. Macroautophagy was originally classified as a non-selective pathway, whereby protein aggregates and dysfunctional organelles are degraded via four steps of autophagic flux: initiation, autophagosome formation, maturation and fusion with lysosomes, and degradation of autolysosome cargo to generate substrates for cellular recycling. Autophagosome formation, a key event in the process, is orchestrated by a large number of proteins, known as ATGs (autophagy-related genes).

In selective autophagy (UPS), targets are specifically marked with ubiquitin before being recognized by autophagosomes via adapter proteins. Based on the nature of the cargo, selective autophagy can be classified as aggrephagy, which targets protein aggregates; mitophagy, which targets damaged mitochondria; peroxophagy, which involves the degradation of peroxisomes; and xenophagy, which involves the destruction of cytosolic pathogens. Rapid degradation of abnormal proteins is the primary role of the UPS, but it is also involved in cell cycle promotion via the sequestering of its inhibitors and acts as a protective response to other stress-mediated events, such as the elimination of oxidized proteins.

CMA is also a form of selective autophagy, independent of vesicle formation, which is completed by lysosomal proteolysis of cargo recognized by chaperone proteins. The main role of CMA is to maintain cellular proteostasis by degrading proteins, protein aggregates and dysfunctional organelles. What is important is the crosstalk between autophagic mechanisms, which is crucial for maintaining homeostasis in cases where a single pathway of the clearance degrading system is insufficient or fails.

Autophagy is crucial for the proper functioning of the central nervous system. In neurons, it clears large and insoluble protein aggregates or damaged organelles and is also essential for synaptic function, which makes neuronal cells particularly vulnerable to dysfunctional autophagy.

Accumulating evidence suggests that impaired autophagy contributes to neurodegenerative disease pathogenesis through several interrelated mechanisms. Although enhanced autophagy is observed in the initial stage of Alzheimer’s disease, as a protective response to stress, disease progression is ultimately accompanied by dysfunction of the autophagic flux. Both macro- and selective autophagy pathways, including the ubiquitin–proteasome system (UPS) and chaperone-mediated autophagy (CMA), are involved in Aβ and tau metabolism, which are important for preventing protein aggregation. Furthermore, the accumulation of Aβ may lead to oxidative damage in mitochondria. However, the malfunctioning organelles are not adequately cleared due to the reduced mitophagy observed in AD patients. Recent evidence indicates that functional autophagy is necessary for synapse functions, including neurotransmission and plasticity. Synaptic dysfunction is also a characteristic feature of AD pathology [32].

Similar to AD, studies of PD pathogenesis indicate that the aggregation of α-synuclein and tau is a result of impaired autophagic–lysosomal degradation, which subsequently impacts mitochondrial functions. Dopaminergic neurons, presenting a high level of metabolic activity, are especially vulnerable to deficits in mitochondrial energy and oxidative imbalance resulting from the accumulation of defective mitochondria. Postmortem analysis has shown evidence of impaired autophagy, chaperone-mediated autophagy (CMA) and mitophagy in the brains of PD patients. A number of genes related to PD are functionally linked to the autophagy pathway. Disease-associated mutations can impair the autophagic flux, while autophagy defects can dysregulate gene expression and downstream signaling pathways. This creates a pathological feedforward loop that exacerbates autophagy dysfunction and impacts associated cellular events that are significant for disease progression [33].

Based on growing body of evidence, dysfunctional autophagy appears to promote proteinopathies associated with ND and subsequently affects neuronal function. Therefore, stimulating autophagic flux is beneficial for mitigating disease progression. Several pathways of autophagy are considered therapeutic targets for both Alzheimer’s disease and Parkinson’s diseases. Moreover, it has been postulated that autophagic mechanisms also decrease in the brain during normal aging; thus, interventions to prevent this phenomenon might reduce the risk of age-related ND [34].

### 2.3. Cellular Senescence

Although the aging brain is prone to the development of neurodegenerative diseases, the mechanism by which senescence within the central nervous system contributes to neuropathogenesis is not clearly understood.

The program of cellular senescence can be initiated in response to a range of stress stimuli. Senescent cells, although viable and metabolically active, are characterized by irreversible cell cycle arrest triggered by the p16INK4a/Rb and p21Cip1/p53 pathways. Senescence activation leads to several molecular changes, including chromatin remodeling, DNA damage response, lysosome enlargement, macromolecular disruption, metabolic disbalance, apoptosis resistance and a senescence-associated secretory phenotype (SASP).

The SASP, a hallmark of senescence, is characterized by the synthesis of various biologically active molecules, such as inflammatory mediators, growth factors and extracellular matrix proteins. These factors reinforce the senescent phenotype through autocrine or paracrine signaling and can also affect the microenvironment, influencing neighboring cells and distant locations within the organism. In contrast to transient physiological states, the chronic persistence of cells presenting a SASP may lead to a snowball effect, ultimately causing serious organ dysfunction underlined by inflammatory damage. As the number of senescent cells increases with age, there is increasing evidence suggesting the involvement of cellular senescence in the pathogenesis of age-related disorders, including neurodegenerative diseases such as AD and PD.

It has been suggested that the phenomenon of cellular senescence may contribute to neuropathogenesis, mainly by promoting chronic inflammation within the central nervous system. Various types of cells within the nervous system have been identified as undergoing the senescence process, including neural stem cells, neurons, astrocytes, oligodendrocytes and microglia. It has been reported that the final dysfunction and neuronal loss observed in ND are often accompanied by malfunctions of other types of central nervous system (CNS) cells, such as microglia and astrocytes [35]. Therefore, the elimination of senescent cells within the CNS, or at least delaying their senescence, and mitigating the negative effects of a spreading SASP have been identified as targets for the prophylaxis and adjunctive treatment of ND.

Cellular senescence can be induced and accelerated by various insults, including oxidative and genotoxic stress, as well as disruption of inflammatory homeostasis. Therefore, protecting cells from harmful external stimuli is considered a way to delay the aging process and alleviate its negative effects at the cellular level. Recent investigations have shown that certain natural compounds with antioxidant and anti-inflammatory properties may be effective in preventing the phenomenon of ‘oxi-inflamm-aging’, which contributes to the neuropathogenesis of ND. Furthermore, the SASP phenotype can be viewed as an ‘inflammatory chain reaction’ that promotes damaging effects. Therefore, biomolecules with antioxidant and anti-inflammatory properties would be beneficial not only as protectors against senescence induction but also as tools to extinguish the spreading fire of inflammation [36].

The signaling pathways activated in senescent cells that promote the SASP could be considered therapeutic targets in ND treatment. The nutrient-sensing pathways, such as mammalian target of rapamycin (mTOR), activated in senescent cells, could be such a target for modulation, as inhibition of mTOR can suppress the SASP [36].

The SASP is also mediated by transcription factors, e.g., nuclear factor κB (NF-κB) and CCAA T/enhancer-binding protein-β (CEBPβ), which subsequently trigger secretion of proinflammatory cytokines (IL6 and TNFa), chemokines, macrophage inflammatory protein (MIP) and transforming growth factor (TGFβ) [37].

Moreover, epigenetic events associated with cellular metabolic activity and mitochondrial status can modulate aging and cellular senescence. Mitochondrial dysfunction, which results in a lower NAD+/NADH ratio, is another cellular incident that has been proven to induce senescence and is associated with aging. This metabolic imbalance affects the function of NAD+-dependent histone deacetylases (HDACs), known as sirtuins. The HDAC class III comprises seven members (SIRT1-7), which have been identified as modulators of cellular senescence and lifespan. Among them, SIRT1 is considered a key regulator of senescence and could come to the rescue as an inhibitor of this process, since sirtuins’ downregulation is associated with aging [38,39,40].

Sirtuins are commonly expressed in the CNS and regulate several pathways related to energy metabolism, mitochondrial function, stress response and damage repair, which makes them vital players in brain aging.

Recent reports have suggested that sirtuins may be involved in the pathogenesis and progression of NDs, although their interrelations need to be elucidated. Similarly to age-related studies, the existing findings indicate the neuroprotective role of SIRT1 in NDs. In AD models, SIRT1 reduces the levels of Aβ and tau phosphorylation, as well as oxidative stress and neuronal loss. In PD models, SIRT1 was found to exert its protective effects by influencing α-synuclein metabolism and other stress response pathways.

As SIRT1 is involved in age-related processes and its downregulation negatively impacts the ND course, sustaining SIRT1 levels seems beneficial in ND prophylaxis, making the protein a promising therapeutic target for epigenetic modulation. Additionally, mitochondrial SIRTs, such as SIRT6, which are important for the antioxidative defense of the brain and involved in the machinery of DNA repair, could be considered positive regulators of longevity and neuroprotection against senescence [38].

Although senescent cells have been found in abundance in the CNS of individuals affected by NDs, it remains unclear whether they are the cause or the result of those diseases. Recent findings suggest that pathological protein aggregates detected in NDs, such as Alzheimer’s amyloid-β and Parkinson’s α-synuclein, can induce or accelerate the senescence of CNS cells through several molecular pathways, including SIRT1 suppression and mitochondrial dysfunction, resulting in increased ROS generation. Cuanalo-Contreras et al. demonstrate that the phenomenon of protein misfolding, aggregation and accumulation is not restricted to disease states but is rather an inherent process of aging [41]. Golde and Miller introduced the hypothesis of proteinopathy-induced senescence, triggered via a self-reinforcing cycle of pro-inflammatory signals and senescence induced by misfolded protein aggregates [42].

Cellular senescence and autophagy are phenomena activated in response to stress accumulation to rescue cells. Recent findings have demonstrated their interplay, but the nature of this relationship remains to be investigated. Autophagy is programmed to clear damaged organelles or abnormal proteins, contributing to maintaining cell homeostasis. Therefore, it should prevent senescence activation and the exclusion of cells from the normal cell cycle. However, studies have shown that autophagy can also boost the senescence-associated secretory phenotype (SASP), which in consequence can promote the spread of senescence [43].

The complexity of cellular senescence and its interrelation with other processes involved in brain aging pose a great challenge. However, each molecular event contributing to this phenomenon may be considered a therapeutic target, creating a chance for multipurpose intervention aimed at minimizing the risk of ND development or slowing down its progression.

### 2.4. Neurogenesis

One of the theories aimed at elucidating age-related cognitive decline claims that the loss of mental abilities happens due to decreased neurogenesis caused by a diminished number and activity of neural stem cells [44,45]. Although neurogenesis has also been detected in other brain regions, it is typically observed at the hippocampal dentate gyrus (in the subgranular zone) and subventricular zone of the lateral ventricles [46,47]. While the presence of neural stem cells in adult human brains is well proven [16], the occurrence of processes like adult hippocampal neurogenesis in humans remains unclear [45,48,49]. Adult human neurogenesis is described as a multistage process comprising quiescent radial glia-like cell activation to generate amplifying neural progenitors, which are then differentiated into neuroblasts. After migration, neuroblasts are developed into immature and mature neurons [50].

The neurogenesis rate drops significantly in early adulthood and then diminishes slowly during the ageing process [49]. Studies performed in rodents have shown that neural stem cells are subject to senescence, secretion of proinflammatory agents and accumulation of protein aggregates—phenomena also characteristic of ND. A decrease in neurogenesis is observed in patients suffering from ND such as AD and it is presumed that the inability of quiescent neural stem cell to activate is the triggering factor of ND [49,51].

The hippocampus, being engaged in memory and emotional processes, is also one of the most severely affected brain regions in AD, resulting in memory loss and the inability to learn—symptoms widely ascribed to AD [52]. However, decreased hippocampal neurogenesis is not limited to AD, as it has been observed in other pathological conditions of the central nervous system, such as PD, major depressive disorder, post-traumatic stress disorder and bipolar disorder, and it is hypothesized that the inhibition of neurogenesis in the dentate gyrus, either by stress or other harmful factors, may lead to pathological changes in processes pertaining to mood, anxiety and cognitive flexibility [50,53,54].

In a study utilizing accelerator mass spectrometry measurement of carbon isotope 14C integrated at the level of cell DNA as a method to retrospectively birth date hippocampal neurons, it was estimated that 700 new neurons are generated and integrated into some of the heterogeneous neuron populations in an adult hippocampal dentate gyrus, which is believed to be enough to affect brain function, cognitive abilities, behavior and well-being [55,56].

In vitro studies conducted on a human hippocampal progenitor cell line have shown that hippocampal neurogenesis is influenced by stress and glucocorticoid hormones. The glucocorticoid receptor (GR), activated by cortisol, increased serum and glucocorticoid kinase 1 (SGK1) expression in human neural stem cells. SGK1 appears to be a mediator of glucocorticoid’s influence on neural cells and plays a vital role in the cortisol-induced decrease in proliferation and differentiation of hippocampal progenitor cells, inhibiting molecular pathways like Hedgehog signaling and sustaining GR activation after cortisol removal [57,58].

Brain-derived neurotrophic factor (BDNF) was observed to promote adult neurogenesis in the hippocampus and its deficit was also observed in other CNS related disorders, such as ND [59].

There is evidence suggesting that the Wnt/β-catenin pathway and non-canonical Wnt signaling is a pivotal player involved in hippocampal neurogenesis, dopaminergic neuron generation and survival in the substantia nigra, while disfunctions in Wnt signaling has been connected to age-related neurogenesis decline observed in neurodegenerative diseases, such as AD and PD [50,60,61]. Up to date, there are 19 members of the Wnt glycoprotein family described that, via activating 10 different Frizzled transmembrane receptors, regulate distinct stages of neurogenesis, specification of neural phenotype and neurorestorative processes [62,63]. The crucial inhibitor of the Wnt/β-catenin pathway is the glycogen synthase kinase-3β enzyme (GSK-3β), as it promotes β-catenin destruction, disallowing its translocation into the nucleus and association with transcription factors controlling genes involved in neurogenesis, neurosurvival and repair. GSK-3β overexpression has been found to inhibit neurogenesis and cause cellular death, while GSK-3β inhibitors are studied as promising therapeutics for neurodegenerative diseases [63,64,65].

Other than neurons, astrocytes have been observed to retain the ability to re-enter the cell cycle and are modulators of hippocampal neurogenesis and neuron maturation [66,67]. Oligodendrocyte precursor cells (OPCs) also have the ability to proliferate and even hasten their cell cycle and differentiation into oligodendrocytes, allowing fast regeneration of myelin in response to injury and aging, as well as adjusting myelination patterns due to learning processes [68]. Again, it has been noted that rodent models of oligodendrocyte generation and myelination processes may not present the according progression of these processes in humans [69]. Microglia have a distinct origin from other neural cells and are not limited in terms of proliferation. The genesis of microglia tends to be triggered by CNS injury; their genesis remains controversial and is suggested to be species- and region-specific [70,71].

Plenty of evidence shows that glia, both astrocytes and microglia, are key factors regulating adult neurogenesis, affecting the process at all stages, from influencing stem cell proliferation to neuron maturation into a specific phenotype and later survival and regulation of synaptic plasticity [66]. For instance, astrocytes can produce molecules such as Wnt1 that cause Wnt/β-catenin pathway activation and both astrocytes and microglia secrete lipocalin-2, a multifunctional protein modulating, among other activities, iron homeostasis, inflammation and cell differentiation. During inflammatory response, activated astrocytes increase lipocalin-2 secretion, further activating astrocytes and triggering reactive microglia, leading to neurogenesis inhibition by arresting neural stem cell (NSC) cycle progression and inducing neuronal apoptosis [72,73,74]. Phagocytic microglia were found to secrete molecules reducing neuronal differentiation and promoting adult hippocampal neurogenesis and there is evidence suggesting that microglia residing in the neurogenic niche present a distinct morphological and transcriptomic profile [49,75]. Moreover, astrocytes, microglia and oligodendrocytes are engaged in lactate homeostasis, including its secretion and delivery to neurons, taking part in neurogenesis modulation [76].

There is another strategy to keep CNS plasticity and regenerating abilities, which seems to be adopted by large-brained mammals, such as humans, besides proliferation of neural stem cells and so-called stem cell-based neurogenesis. Immature neurons have been found in post-mortem brains of elderly people; however, their number appears to diminish with age. So instead of continuous production of new progenitor cells, prolonging the neural cell maturation period is favored, keeping the immature neurons dormant as new cells supply the brain [77].

The Notch signaling pathway, suppressed by pro-neurogenic miR153 in mice and humans, as well as Hedgehog signaling, are involved in keeping NSCs in their quiescent state, while the Wnt pathway molecules appear to trigger NSC differentiation and maturation [78].

**Figure 3:**
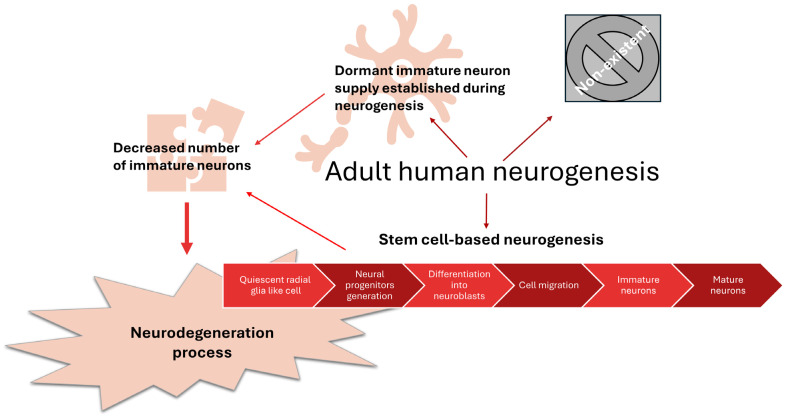
Neurogenesis involvement in ND pathogenesis. The theories regarding adult human neurogenesis remain inconclusive as the current state-of-the-art does not allow non-invasive and precise enough observation of the process occurring in the human CNS. Since a decrease in immature neurons in ND patients has been noted, a block in the neurogenesis process is hypothesized to underlie the pathogenesis of ND.

The theories regarding neurogenesis and its involvement in ND pathogenesis are illustrated in Figure 3.

### 2.5. Blood–Brain Barrier Dysfunction

**Figure 4:**
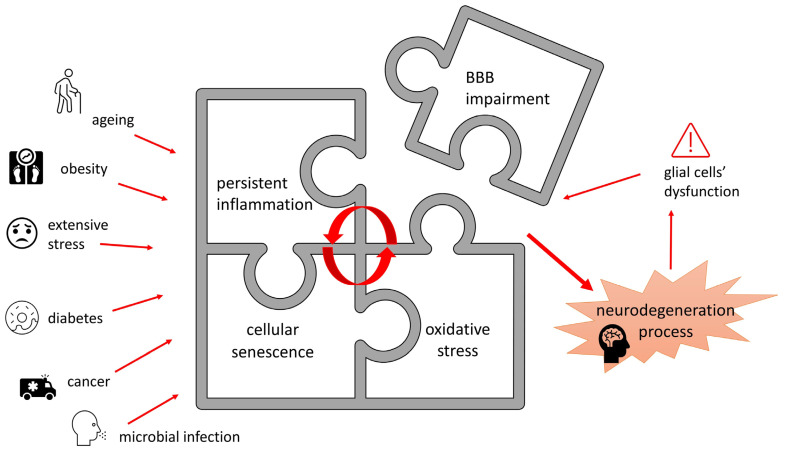
The interrelation of BBB permeability and processes involved in ND pathogenesis. BBB impairment is involved in ND pathogenesis via several complex mechanisms driving neurodegeneration. Environmental factors can also trigger processes endangering BBB integrity, such as oxidative stress, inflammation, cellular senescence and SASP and pose further threat to CNS cells which, unprotected by a compromised BBB, are vulnerable to harmful agents present in the systemic circulation.

Another structure implicated in neurodegeneration processes is the BBB, a monolayer of unique, tightly packaged microvascular endothelial cells and the hydrophobic basement membrane, surrounded by pericytes and adjoining astrocyte feet. It lines the cerebral blood vessels, limiting paracellular and transcellular transport to the CNS by creating tight junctions and allowing the paracellular passage of any particles bigger than 800 Da to occur only by means of specialized transporters, strictly regulating compound exchange between the circulatory system and brain parenchyma [79]. As the BBB is dynamic, reactive to signals from the endocrine system structure, it not only limits the entry of neurotoxic substances and immune cells circulating in the blood flow to the CNS, but also responses to the metabolical demands of the CNS by regulating nutrient intake, expelling metabolic waste and acting as an endocrine tissue releasing various local hormones, including cytokines [80,81]. BBB dysfunction appears to coexist with, or even precede, major CNS neurodegenerative conditions, such as AD, PD and multiple sclerosis. Though the primary cause is often still unknown, it is supposed that the malfunction of a single class of cells creating the barrier is enough to render the whole structure faulty. A leaky BBB introduces or exacerbates neuroinflammation in the CNS. However, it is also observed that neuroinflammation is the cause of increased permeability of the BBB [80,82,83]. Moreover, inflammation triggers BBB endothelial cells to release extracellular vehicles that contain proteins enabling the migration of circulating T-cells into the CNS, therefore compromising the BBB [82]. The simplification of BBB involvement in ND pathogenesis is presented in Figure 4.

While BBB impairment is often associated with age, systemic inflammation, extensive stress, obesity, diabetes, cancer and microbial infection are recognized as factors further increasing the risk of heightened BBB permeability [84,85,86]. There are hypotheses ascribing ND incidence to age-associated BBB dysfunction. Moreover, the barrier provides varied protection to different brain structures, due to vascular system diversity. The cortical regions are less vulnerable to harmful agents present in systemic circulation than the hippocampus formation, due to increased BBB permeability in that region [86,87]. Age-related decline in BBB integrity and its transportation functionality is supposed to be a major risk factor in the pathogenesis of AD and memory deficits [86,88]. Interestingly, a large body of evidence suggests that the accumulation of pathological proteins such as amyloid-β, hyperphosphorylated tau or α-synuclein, characteristic for the most common NDs, significantly disrupts the BBB, which in turn leads to damage to neural cells [89].

However, it is hypothesized that BBB deterioration caused by aging is not enough alone to trigger ND; an occurrence of another detrimental factor, for instance systemic inflammation affecting astrocyte and pericyte interactions with endothelial cells, as well as microglia malfunctioning, would be necessary.

Microglia are involved in BBB endogenous repair mechanisms and appear to regulate BBB permeability by directly interacting with endothelial cells and releasing proinflammatory cytokines in response to various disrupting conditions. Astrocytes produce factors activating Wnt signaling in endothelial cells, crucial for maintaining BBB integrity. These interactions are disrupted in age-associated pathologies, for instance in PD, repression of Wnt signaling in astrocytes concomitant with microglial inflammatory activation, as well as BBB leakage, were noted [90,91].

## 3. Probiotic By-Products as Tools Supporting CNS Function

According to the Food and Agriculture Organization and the World Health Organization, the definition of probiotics is formed as “living microorganisms that when administrated in adequate amount confer a health benefit on the host” [92]. The term probiotics applies mainly to lactic acid bacteria (LAB), Bifidobacterium spp. and yeast [93]. The application of probiotics demonstrates beneficial effects like competitive exclusion of pathogenic microorganisms and secretion of antimicrobial substances, enhancement of the epithelial barrier function, production of short-chain fatty acids (SCFAs) and other metabolites, restoration of the homeostasis of the intestinal microbiota and immunomodulatory activity [94,95]. On the other hand, many researchers have investigated the properties of inactivated or non-viable microorganisms, termed paraprobiotics [96]. Their beneficial effect has been observed even when non-viable bacteria or fragments of cells are used. In contrast, postbiotics are metabolites, soluble factors secreted by beneficial microorganisms or even cell-free supernatants, which have a beneficial impact on host cells [96]. Postbiotics have immense potential due to their dual source—from the outer elements of bacterial cells and from inside of them. The primary elements of bacterial cells are polysaccharides, peptidoglycans, lipoic acid, phosphonic acid, cell surface proteins, cell membrane proteins and extracellular polysaccharides. The secondary metabolites of probiotic bacteria are short-chain fatty acids, vitamins, proteins and enzymes, organic acids like propionic acid and 3-phenyl lactic acid, and intracellular polysaccharides [94,97]. Since many different particles are included in the term postbiotics, they have many beneficial properties like bacteriostatic activity, immunoregulation, antioxidant activity, liver protection, blood pressure-lowering activity, regulation of intestinal flora and prevention and treatment of constipation, enteritis, and other diseases [97]. Looking broadly, postbiotics have many benefits, such as being safer and more predictable than probiotics, so they can address a broad range of problems. The application of postbiotics does not result in any risk of infection for sensitive or immunocompromised groups of patients, like newborns or people with a low immune response (weak immune system). Moreover, their application with antibiotics lacks the risk of transmitting resistance genes to another microorganism [95]. Postbiotics are more stable, since no living organisms are inhabiting the mixture; therefore, they demonstrate higher resistance to oxygen, temperature, and other environmental influences, so the active factors are not easily destroyed [95,97].

Several in vivo investigations prove a positive influence of probiotics on age-related processes in the context of CNS function. This provides a foundation for studying the pro-health potential of their metabolites and macromolecules at the cellular and molecular level of a host organism [98,99,100,101]. As reviewed by Tsai et al., supplementation with probiotic bacteria of several *Lactobacilli* spp. has been shown to have a positive effect on animal models. An anti-aging effect was achieved through the downregulation of pro-inflammatory cytokines (TNF-α, IFN-γ and IL-1β) and other inflammation markers (*p*-p65, COX-2 and iNOS), as well as the stimulation of antioxidant defense (upregulation of the Nrf2 pathway resulting in the expression of glutathione reductase and glutathione S-transferase; activation of superoxide dismutase and glutathione peroxidase). Additionally, the effect was mediated through the modulation of several cellular pathways, such as the suppression of senescence molecular markers (p16, p53), sustaining of mitochondrial function (respiratory chain, membrane potential and permeability transition), and increasing their number by upregulation of the expression of PGC1α, SIRT1, NRF1 and TFAM involved in mitochondrial biogenesis. What is more, the reduction in telomere shortening, stimulation of BDNF expression and phosphorylation of cAMP-response element-binding protein (CREB) was observed along with the inhibition of aging-associated activation of Akt, mTOR and FOX3a [100].

The probiotic secretome has multipotent bioactivity targeting distinct aspects of cellular senescence and ageing-related processes. It contains numerous molecules that have been shown to counteract molecular events involved in CNS senescence and the pathogenesis of NDs [102]. The pleiotropic pro-health effect of postbiotics makes them a promising tool for ND prophylaxis and an adjunct to standard treatment. Various antioxidant compounds have already been investigated in recent years for their neuroprotective effects, both of natural and synthetic origin. These have mainly been agents from the group of polyphenols, carotenes and vitamins and even hormones [103,104,105]. The main components with antioxidant and radical scavenging activity in postbiotics are bacterial secondary metabolites of phenolic compounds, exopolysaccharides, enzymes such as glutathione peroxidase or teichoic and lipoteichoic acids, which are the components of bacterial cell walls [106,107,108,109,110].

The original and most obvious site of both pro- and postbiotic action, the intestines, is a pivotal player in maintaining host’s homeostasis. Intestinal inflammation has been implicated in a wide variety of diseases, including ND. Increased permeability of intestinal barrier endangers the whole organism by letting ingested pathogens and other harmful agents pass into systemic circulation and influence a variety of organs. Moreover, depleting intestinal microbiota, a key factor in the synthesis and metabolism of plenty of beneficial substances for both the host and other favorable bacteria, may cause both dietary insufficiencies and the risk of pathogenic microbial infection. For instance, butyrate, one of the microbial metabolites, was found to have a direct influence on intestine epithelium proliferation, maturation and metabolism, promoting intestinal barrier integrity, suppressing inflammation and preventing cancerous transformation [111].

Additionally, one of the theories aimed at elucidating PD pathogenesis, Braak’s hypothesis, claims that the disease starts in the gut, from where misfolded α-syn proteins spread to the brain by the vagus nerve. So far, there are both in vitro and in vivo results, obtained from both animal and human models, that appear to be in accordance with this idea [112].

Shortly speaking, a well-balanced microbiota is necessary for intestinal barrier proper functioning, and the importance of the intestinal barrier’s fitness for maintaining the health and well-being of the whole organism cannot be overestimated [113]. However, since ageing has been correlated with an abundance of harmful bacteria producing pro-inflammatory cytokines, such as enterobacteria, streptococci and staphylococci, together with a deficit in the healthful *Lactobacilli* and *Bifidobacteria*, preserving diversified, robust microbiota may require some effort, but appears to be an auspicious option for ND prophylaxis [114].

Another barrier involved in ND pathogenesis, which is also influenced by microbiota composition and its metabolites that have traversed the abovementioned intestinal barrier, is the BBB. Improving BBB integrity has long since become a therapeutic target in ND therapy. Apart from synthetic drugs, such as the anticancer medicament Axitinib or the microglia inhibitor Minocycline, compounds of natural origin have been taken under investigation, with a sharp focus on microbial metabolites. Gut dysbiosis and alterations in gut microbiota, and therefore a change in microbial metabolite secretion, have been proven to affect the CNS and play a significant role in ND pathogenesis [90]. Applying antibiotics causes a pronounced alteration in microbiota composition with a significant depletion of SCFAs producing bacteria, as well as increased BBB permeability, a change in tight junction protein expression and even mood and cognitive impairment in animal models [90].

Moreover, it was observed that disruption of the gut microbiota in mice by antibiotic therapy causes a significant decrease in adult hippocampal neurogenesis and alters gene expression in the hippocampus, upregulating genes involved in apoptosis and immune responses, including the pro-inflammatory response, while triggering behavioral deficits like anxiety and spatial memory and social behavior impairment. Healthy microbiota transplantation reversed behavioral disorder, decreased pro-inflammatory gene expression and restored neurogenesis. The suggested modulators of these processes are serum metabolites produced by gut bacteria, 139 of which were significantly changed between the microbiota-disrupted and undisrupted mice, including bacteria-derived lipids that are reported to cross the BBB [34]. A total of 30 days of oral administration of *B. longum* and *B. bifidum* to an AD transgenic mice model reduced the death of hippocampal neurons and restored BDNF levels and synaptic scaffolding proteins, causing a decrease in Aβ42-positive cells and proinflammatory response, as well as mitigating cognitive decline triggered by the proteinopathy [115].

### 3.1. Postbiotics as Mixture of Molecules Targeting Cellular Machinery of ND Pathogenesis

The impact of probiotic seretome on CNS function has been initially investigated using neural in vitro cultures or animal models treated usually with the post-culture supernatants derived from bacteria cells. This experimental approach has revealed a wide range of beneficial effects of this complex mixture of microbial metabolites on the function of CNS cells. *Parabacteroides distasonis*- and *Megasphaera massiliensis*-derived postbiotics were chosen from a panel of 50 gut microbiota-produced mixtures for their strongest neuroprotective properties. Not only were the supernatants providing antioxidant activity, but they also promoted the differentiation of neural cells and decreased pro-inflammatory cytokine secretion in human microglia, and they also preferentially protected differentiated neurons from oxidative stress. The mixture’s cytoprotective qualities were mainly ascribed to SCFA, indoles, lactate and succinate. It is worth noting that applying SCFA alone to the cells did not replicate the results achieved for postbiotic mixtures [116].

Moreover, soy milk fermented with *L. plantarum* with the addition of oligosaccharides had a neuroprotective effect on H_2_O_2_-treated cells, increasing their viability, BDNF and tyrosine hydroxylase expression, while decreasing proapoptotic gene expression [117].

Post-culture media produced by lactic acid-producing bacteria, comprising *Bifidobacteria* and *Lactobacilli*, were also observed to protect neural cells from the cytotoxic influence of in vitro ND inducers: rotenone and MPP+ [118]. A post-culture mixture of *Bifidobracterium longum* increased BDNF expression in corticosterone-stimulated SH-SY5Y cells [119].

Apart from neuroprotective potential, the cell-free supernatant obtained from the selected LAB strains exhibited an anti-senescent effect at the cellular level, confirmed also on an in vivo animal model of induced premature senescence [120].

The promising results proving both the direct and indirect neuroprotective potential of postbiotics created the background for further investigation aimed at elucidating their influence on cellular events involved in the function of CNS cells and the pathogenesis of neurodegenerative disorders.

#### 3.1.1. Short-Chain Fatty Acids

SCFAs are the most extensively investigated group of postbiotics in the context of their neuroprotective potential, with butyric acid being the rising star [100]. Butyrate production impairment observed in ND patients is linked to the disease pathogenesis and occurring symptoms, as the molecule’s multifunctional potential is still being uncovered. Up-to-date, butyrate has been found to exert beneficial influence on, among other activities, cellular metabolism, apoptotic processes, physiological barrier integrity, microbiota composition, epigenetic modifications, inflammation, cell growth and differentiation and cell cycle progression [121].

It has been shown that levels of SCFAs decline with age, which may indirectly contribute to perturbations in CNS function, e.g., via the disturbance of neural microglia maturation and function.

On the other hand, SCFA supplementation has resulted in overall improvement in terms of inflammation, neural health and aging. Due to the molecular basis of their action in relation to cellular senescence, SCFAs have antioxidant and anti-inflammatory potential, evidenced in vitro and in vivo experiments [122].

**Figure 5:**
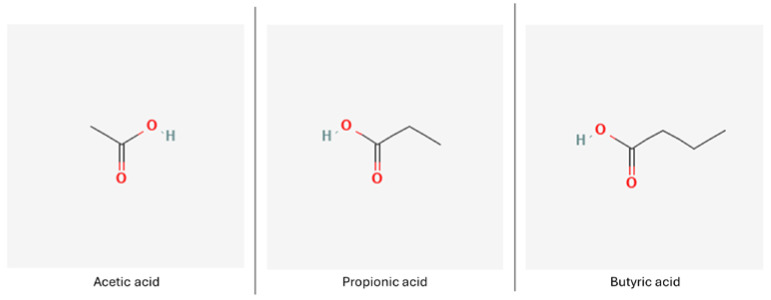
Short-chain fatty acids. Acetic acid (PubChem 176 [125]), propionic acid (PubChem 1032 [126]) and butyric acid (PubChem 264 [127]).

SCFAs such as butyrate, acetate and propionate (Figure 5) also function as histone deacetylase inhibitors (HDACis), which can act on the epigenome through chromatin remodeling changes [123,124].

An increase in SCFA production, caused by probiotic supplementation, raised serum levels of SCFAs that then crossed the BBB in higher concentrations and suppressed GSK-3β activity in the CNS, alleviating ND pathology [128,129].

SCFAs and the presence of their receptors were also reported to be crucial for adult neurogenesis in both wild-type and AD model mice [130]. Increased SCFA production correlated with increased BDNF serum levels in ADHD patients [131].

Notch, Hedgehog and Wnt pathways are affected by SCFAs, which have been widely researched in cancer studies. Neoplastic cells appear to lose their “stemness” and undergo differentiation processes upon SCFA, especially butyric acid, treatment. Such treatment of the undifferentiated “back-up” neurons, according to one of the hypotheses regarding human adult neurogenesis, would replenish injured neurons and alleviate ND-inflicted cognitive decline. The mechanisms underlying SCFA action are attributed to their causing epigenetic alterations such as HDAC inhibition, DNA methylation pattern changes and histone phosphorylation [132].

So far, experimental data suggests that SCFAs impact astrocyte activation and molecules secreted by astrocytes in turn affect OPC proliferation [133].

Long-term SCFA supplementation in AD-like mice influenced intestinal microbiota composition, reduced Aβ and tau proteinopathies and ameliorated cognitive deficits. Single-cell sequencing data suggest that the phenomenon stems from SCFAs stimulating the glutamate–glutamine shuttle, exerting antioxidant effect on CNS cells and promoting astrocyte–neuron communication in the hippocampus. SCFAs were able to alleviate oxidative stress-induced damage to neurons and protect them from mitochondrial injury by supporting astrocyte functioning [134].

Propionate and butyrate were found to have a protective action toward BBB cells, ensuring BBB integrity and homeostasis [89]. SCFAs, especially butyrate, as well as a LAB-derived mixture, also promoted BBB restoration after an injury inflicted by LPS administration—microbiota antibiotic depletion caused by antibiotics. SCFAs’ beneficial effect on BBB epithelial cells was also observed in mouse ND models [85].

#### 3.1.2. Lactate

**Figure 6:**
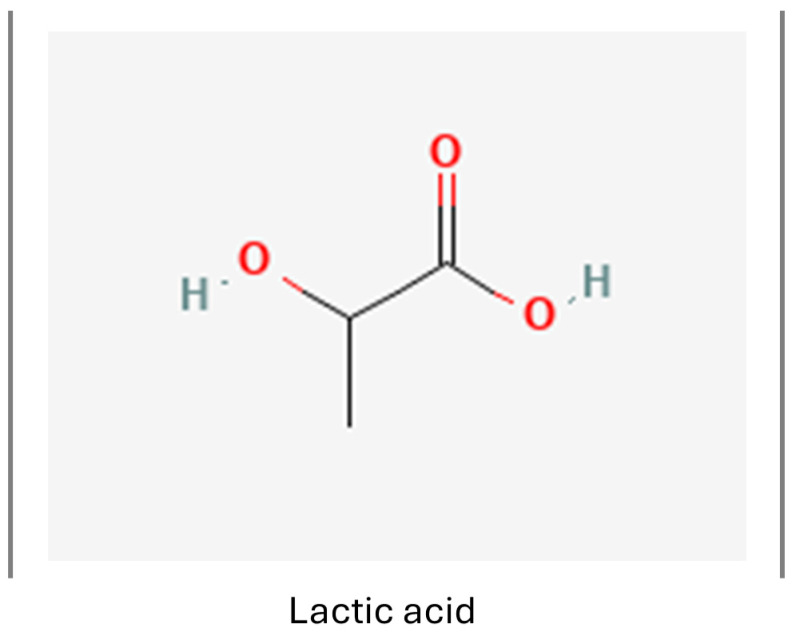
Lactic acid (PubChem 612 [138]). Conjugate acid of lactate.

Lactate, a salt of lactic acid (Figure 6) found in the CNS, is not only produced by astrocytes, but also LAB bacteria or physical activity, and it crosses the BBB via several monocarboxylate transporters. It plays a pivotal role in brain pH determination and can be used as an energy source by neural cells. It has been recognized for its neuroprotective effect and ability to promote neurogenesis in murine models by activating the NF-κB signaling pathway [135,136]. Moreover, lactate was observed to promote OPC differentiation [137].

However, excessive extracellular lactate accumulation has been reported to inhibit hippocampal neurogenesis, highlighting the importance of maintaining lactate homeostasis by endothelial cells and astrocyte transporters such as MCT1, which is also involved in SCFA transportation through the BBB [139].

Histone lactylation, a post-translational addition of the lactyl group at lysine sites of histones, is a part of epigenetic coding that has been recently uncovered and quickly gained attention in regard to neurodegenerative processes. There is evidence showing extra- and intracellular levels of lactate stimulating histone lactylation, which in turns impacts gene expression and immune system functioning [140]. The sole process of histone lactylation and its interactions with other epigenetic mechanisms remain to be elucidated and are widely studied in the context of various disorders’ progression, such as bacterial and parasitic infections and cancer and tumor microenvironments [141]. AD patients’ sequencing results have shown an abundance of lactylation at the H4K12 site in microglia, which is believed to trigger considerable alterations in metabolic pathways, involving glycolysis, and specifically upregulate pyruvate kinase expression. This created positive feedback loop results in aggravating inflammation and fans the flames of disease progression [142]. Moreover, H3K18 lactylation occurring on senescent microglia in mice exacerbated AD. An increased level of lactylation was observed in both naturally aged and AD model mice in microglial cells and hippocampus tissues. The epigenetic modification stimulated IL-6 and IL-8 expression stemming from NFκB signaling pathway activation by increasing binding to the promoters of its two activators, Rela and NFκB1, consequently promoting SASP advancement [143].

On the other hand, exercise-derived lactate caused microglia to switch to an anti-inflammatory phenotype and alleviated cognitive decline in AlCl3/D-gal-treated mice. In this study, the exercise-produced lactate was simulated by intraperitoneal injection of sodium lactate. Aβ_1-42_ treatment stimulated BV2 cells to secrete lactate and the addition of sodium lactate increased histone H3 lactylation. Acetyltransferase p300 knockout was able to reverse the process and trigger anti-inflammatory gene expression, demonstrating that p300 has a mediating effect on this phenomenon [144].

As has been highlighted frequently in the current literature, the phenomenon of histone lactylation is not yet comprehensively understood and requires further investigation. Recently, it was proposed that lactate regulates the mechanism of the microglia anti- or pro-inflammatory phenotype switch as a sort of epigenetic accelerator for the timer that modulates the microglial activation and subsequent shift into repair mode [144].

#### 3.1.3. Polyamines

Although polyamine level is strictly controlled, it dwindles with age, and it was reported to be significantly decreased in NDs such as AD and PD [145]. Polyamine dysregulation, the hypothesized effect of a prolonged polyamine stress response which can be triggered by physiological and emotional stimuli and alters central polyamine homeostasis, was observed in tauopathies, including the forementioned AD, and is thought to be induced by the chronic stress inflicted by tau accumulation. The dysregulation further aggravates pathophysiological conditions and resulting cognitive decline, as acetylated polyamines produced by spermidine/spermine-N1-acetyltransferase, a part of the enzymatic regulatory machinery, stimulate tau fibrilization [146].

While polyamines are present in both plant and animal products, fermented food was found to contain the highest levels of said biomolecules, as the role of microorganisms in producing polyamines is undoubtedly substantial [147]. Bacteroides and Fusobacteria, although not members of the LAB group, are among the most represented genera in the human gut. They were reported to produce copious amounts of polyamines: spermidine and spermine in both in vitro and in vivo conditions [148]. Some of *Lactobacilli*, f. e. *L. hilgardii* X1B and N4 strain of *L. plantarum*, were also proven to synthetize polyamines [149].

Spermidine was observed to influence a wide range of cellular processes, including proliferation, differentiation, autophagy and senescence. It also exerted antioxidant effects on various cellular models [148]. Spermine and spermidine anti-aging effects were also reported on senescence-accelerated mice. The phenomenon was ascribed to polyamines’ antioxidant and autophagy mediating properties, as well as their ability to upregulate neurotrophic factors, balance mitochondrial function and inhibit proinflammatory proteins expression [150].

Spermine protected dopaminergic neurons of α-syn expressing *C. elegans* (strain UA44) from ND to a considerable degree and alleviated degeneration caused by manganese exposure, which is the origin of manganese-induced PD. The cytoprotective effect against manganese-induced toxicity was also confirmed on SK-MEL-28 cells expressing α-syn [151].

Clinical trials regarding the influence of spermidine intake on cognitive functions by the elderly show inconclusive results. One year of daily supplementation with 3.3 mg of spermidine significantly improved dementia patients’ memory performance, while another study reports that supplementing elderly adults with subjective cognitive decline in a randomized, double-masked, placebo-controlled trial with 0.9 mg of spermidine, 0.5 mg of spermine and 0.2 mg of putrescine per day did not result in significant changes in memory functioning and other behavioral indicators compared to a placebo group. However, as a beneficial effect on verbal memory and inflammation processes is implicated, further research, including dose increase, is planned [151,152,153,154].

#### 3.1.4. Tryptophan Derivates

Tryptamine, indole propionic acid, indole butyric acid and indole derivatives are tryptophan microbiota-derived metabolites that have been observed to influence the aging process and ND development. Only 1–2% of consumed tryptophan is converted to serotonin and melatonin in the serotonin pathway, as most of it is degraded to kynurenine in the kynurenine pathway. Tryptophan metabolism is influenced by many factors, including stress, aging, microbiota composition and inflammatory status [155]. Tryptophan is one of the major substrates for gut microbial metabolism that occurs in multitude of species occupying the colon, including LAB [156].

**Figure 7:**
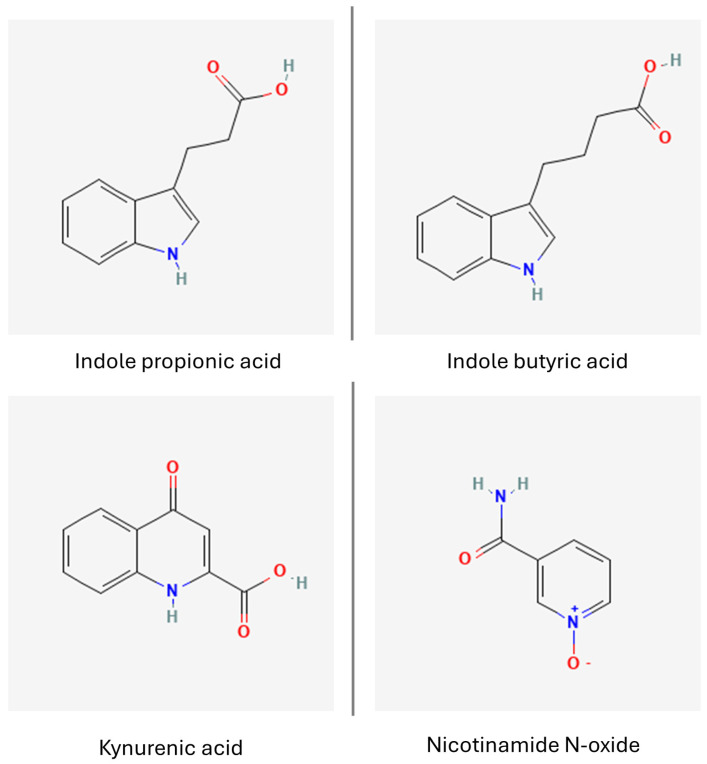
Tryptophan derivatives. Indole propionic acid (PubChem 3744 [163]), indole butyric acid (PubChem 8617 [164]), kynurenic acid (PubChem 3845 [165]) and nicotinamide N-oxide (PubChem 72661 [166]).

Various metabolites, such as indole, indole acetic, propionic and lactic acid (Figure 7) exert neuroprotective properties, causing a considerable reduction in oxidative stress, neuroinflammation and apoptosis in mouse hippocampal neurons injured by H_2_O_2_. Moreover, indole acetic acid and indole propionic acid activated the GPR30/AMPK/SIRT1 pathway in d-galactose-aged mice, hampering the neurodegeneration process [157]. Indoles, produced in the host organism solely by bacteria as humans lack the necessary enzymatic machinery, as well as various other tryptophan metabolites, bind to the aryl hydrocarbon receptors that are expressed in a wide diversity of cells including gut epithelial cells and CNS cells, serving as a mediator molecule between the host and their microbiota [156]. Indoles were noted to stimulate adult neurogenesis by interacting with the aryl hydrocarbon receptors and increasing β-catenin, Neurog2, and VEGF-alpha expression in mouse hippocampal tissues [158]. Other tryptophan metabolites were noted to act as an activator for metalloproteinases, which are responsible for clearing misfolded and aggregated proteins in the brain. 5-hydroxyindole-acetic acid and kynurenic acid (Figure 7) stimulated the expression of neprilisin, a metalloproteinase involved in Aβ degradation, which alleviated the insult caused by Aβ. It is worth noting that neither 5-hydroxyindole-acetic acid nor kynurenic acid are able to pass through the BBB; however, it is suspected that during neurodegenerative conditions, the increased permeability of the BBB would allow them access to the brain [159].

Nicotinamide N-oxide (NAMO, Figure 7) can be produced by the enzymatic transformation of a tryptophan kynurenine pathway product—nicotinamide adenine dinucleotide—but is also a gut bacteria metabolite generated mostly by Lactobacilli. While its levels were observed to fluctuate during infection, gut microbiota-derived NAMO was noted to diminish HSV-1-induced neuroinflammation by inhibiting microglia polarization, promoting a switch to an anti-inflammatory phenotype and restoring mitophagy, contributing to preserving neurons affected by HSV in infected mice. Moreover, NAMO was found to decrease glial pro-inflammatory cytokine release, while enhancing sirtuin 1 expression in BV2 cells [160,161].

On the other hand, kynurenine metabolites such as anthranilic acid and its derivatives, hydroxykynurenine, quinolinic acid and picolinic acid, have been associated with increased oxidative stress, neuroinflammation and neuronal apoptosis and are implicated in a wide spectrum of CNS conditions, including autism, depression and neurodegeneration [156]. Therefore, a careful design of probiotic bacteria composition and culture conditions would be necessary to achieve desired therapeutic results. For instance, *L. reuteri* was observed to preferentially synthesize kynurenic acid, a tryptophan metabolite with healthful effect on the CNS and antioxidant properties, from kynurenine when cultured in Hank’s Balanced Salt Solution, which makes it a viable candidate for producing postbiotics that could be implemented in ND therapy [162].

#### 3.1.5. Polyphenol Metabolites

Gut bacteria are a crucial factor in transforming dietary polyphenols into bioactive molecules that can enter systemic circulation and, to varying extents, penetrate the BBB to exert a beneficial effect on the brain and practically the entire host’s body. In turn, dietary polyphenols modulate microbiota composition and can stimulate the proliferation of probiotic bacteria, while suppressing the spread of pathogenic microbes [167,168].

**Figure 8:**
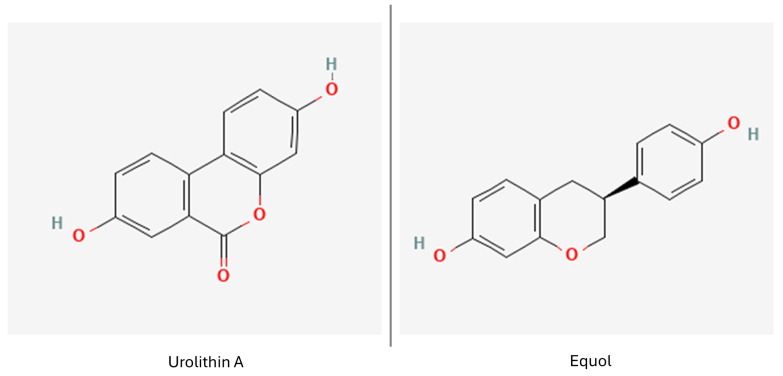
Polyphenol metabolites. Urolithin A (PubChem 5488186 [174]) and equol (PubChem 91469 [175]).

Urolithin A (Figure 8), a bacterial metabolite of ellagic acid, a polyphenol common in vegetables and fruits, has been noted to not only protect BBB integrity, and exert an anti-inflammatory effect on a human neural cell line, but also to promote neurogenesis in an ischemic stroke mouse model. It has recently been under investigation as a potential ND therapeutic [90,169,170]. It has a high ability to cross the BBB and was reported to decrease neuroinflammation in an AD in vitro model, as well as stimulate microglial phagocytosis and mitophagy [171].

Dihydroxyphenyl-γ-valerolactones, bacterial metabolites of catechins and epicatechins, were observed to cross the BBB and promote neuritogenesis, while aryl-γ-valerolactones were shown to exert a beneficial effect on a mouse AD model, stopping amyloid-β oligomerization and altering preformed oligomers into non-toxic forms [171,172].

Equol (Figure 8), a gut microbial metabolite of daidzein, was observed to cross the BBB and exert anti-inflammatory effect on microglia in a murine model, as well as alleviate LPS-induced, 6-OHDA- and MPP+-induced cytotoxicity in SH-SY5Y cells. Its cytoprotective properties against MPP+ were also confirmed in *C. elegans* [173].

#### 3.1.6. Exopolysaccharides

**Figure 9:**
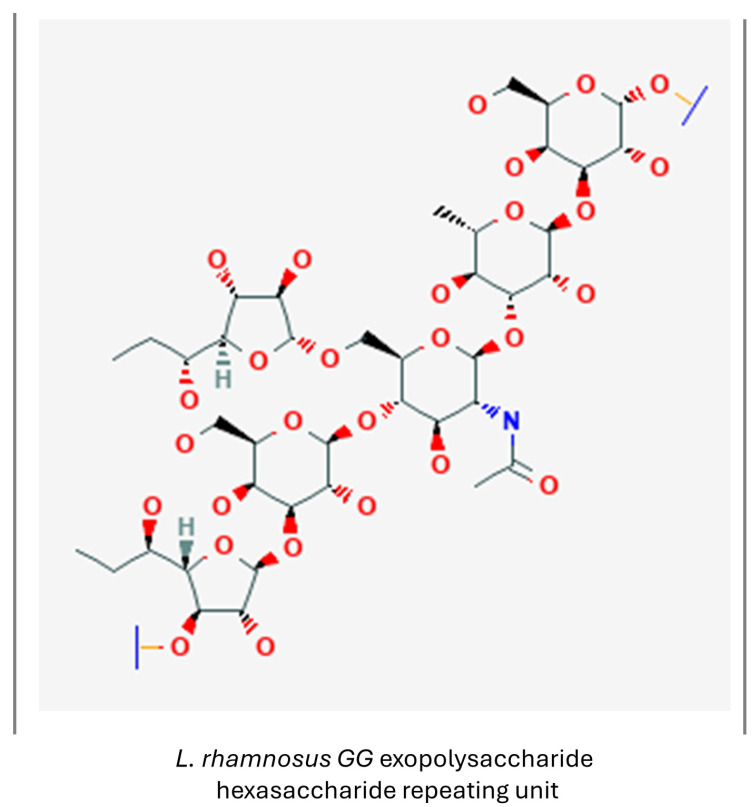
*Lactobacillus rhamnosus* GG exopolysaccharide hexasaccharide repeating unit (PubChem 433987232, [176]).

Exopolysaccharides (EPSs) are high-molecular-weight carbohydrate polymers, extensively produced by LAB. They show high antioxidant activity and are recognized for their anticancer, anti-inflammatory and macrophage-activating abilities. The antioxidant activity of EPSs is thought to result from an increase in the activity of antioxidant enzymes, and a reduction in serum lipid peroxidation [110]. An example of a LAB-produced exopolysaccharide is presented in Figure 9.

In vitro, EPCs isolated from *L. plantarum* were found to protect PC-12 cells from oxidative stress, while EPCs isolated from other LAB cultures, namely from *L. delbrueckii* ssp. *Bulgaricus* B3 and *L. plantarum* GD2, were reported to increase the viability of SH-SY5Y cells injured with Aβ1-42 by alleviating oxidative stress induced by the amyloid treatment [177,178].

In vivo, EPSs as high-weight macromolecules can exert a rather indirect impact on CNS cells, such as an exopolysaccharide produced by *L. rhamnosus* Ram12 exhibiting protective activity against D-galactose-induced brain injury via increasing the level of antioxidant enzymes and total antioxidant capacity, as well as the level of anti-inflammatory cytokine IL-10, while decreasing the level of oxidative stress and pro-inflammatory markers [179].

The well-proven antioxidant properties and capacity to reduce neuroinflammation status make LAB-derived exopolysaccharides a promising neuroprotective agent for the prophylaxis and supportive treatment of ND [180].

#### 3.1.7. Extracellular Vesicles

Extracellular vesicles (EVs) secreted by probiotic bacteria are a type of postbiotic which is receiving increasing attention. Bacteria of all classes produce extracellular vesicles, which is an evolutionarily conserved mechanism for intercellular communication [181]. These vesicles are made of the outer membrane of the bacterial cell and part of cytoplasm inside. Depending on the type of bacteria, Gram-positive or Gram-negative, the construction of the membrane of EVs differs. Gram-negative bacteria’s cell walls are built of two membrane bilayers with a peptidoglycan layer in between. On the outer membrane, various membrane protein channels and leaflets are placed, along with lipopolysaccharides (LPSs), which are endotoxins [182,183]. Gram-positive bacteria have just one inner membrane and a much thicker peptidoglycan cell wall, which are connected by lipoteichoic acids (LTAs). EVs produced by Gram-negative bacteria are built of outer membrane or both membranes, while EVs produced by Gram-positive bacteria are built solely of inner membrane. Both EVs contain membrane proteins and other membrane compounds as well as peptides, metabolism products, DNA and RNA fragments and other particles present in the cytosol [181,182,183]. As EVs are packaged with many bacterial molecules, bacteria communicate with host cells by releasing them. There is evidence proving that bacterial EVs produced by gut microbiota cross the epithelial barrier and enter systemic circulation or the lymphatic system, and then cross the blood–brain barrier and interact with CNS cells directly [182,184]. The LPS present on the surface of LAB EVs can engage the Toll-like receptor 2 (TLR2) [185]. Toll-like receptors (TLRs), as immune receptors present on multiple cell types, are appointed as the mediators on the gut–brain axis. Their dysregulation has been observed in neurodegenerative conditions. TLRs conduct the inflammatory response produced upon their contact with various endo- and exogenous molecules. There is evidence that TLR-mediated neuroinflammation occurs both in AD and PD and while it may be the cause of neuronal loss in PD, the activation of TLR can stimulate α-syn and amyloid-β clearing [186,187,188].

Extracellular vesicles derived from *L. plantarum*, administered to a mouse hippocampal cell line treated with another glucocorticoid hormone—corticosterone—were able to upregulate BDNF expression and alleviate stress-induced depressive-like behavior in mice treated with corticosterone [189]. *L. rhamnosus*-derived EVs inhibited pro-inflammatory polarization of murine microglia treated with LPS and ameliorated the inflammatory response. [190]. *L. paracasei*-derived EVs were able to reverse some of the effects of Aβ-induced pathology on the mouse hippocampus-derived cell line HT22, restoring multiple gene expression, including neurotrophic factors, Aβ-degrading proteases and sirtuin 1, possibly by epigenetic regulations. This effect was also observed in vivo in Tg-APP/PS1 mice. Moreover, EV treatment reduced astrogliosis, increased hippocampal neurogenesis and alleviated cognitive decline in tested animals [191].

On the other hand, pathogenic bacteria also secrete extracellular vesicles, which have a detrimental influence on the host’s well-being. For instance, it is hypothesized that ND occurrence is promoted by dental caries. There is some evidence suggesting that oral pathogens, such as *Aggregatibacter actinomycetemcomitans*, a periodontopathogenic bacteria, secrete vesicles that contain RNA-stimulating pro-inflammatory cytokine expression. Applied in a gel or intragingivally, injected vesicles were able to pass through the BBB and trigger IL-6 and TNF-α release in the brains of ligature-induced periodontal disease mice by activating the TLR4 and TLR8 pathways [192]. Another periodontopathogen, *Porphyromonas gingivalis*, and its extracellular vesicles, are also reported to trigger inflammation and are implicated in dementia pathogenesis. However, the application of *L. pentosus* NK357 and *B. bifidum* NK391 resulted in reducing behavior suggesting cognitive impairment, as well as decreasing the TNF-α level in hippocampal tissues and alleviating neuroinflammation, diminishing periodontitis and gut microbiota dysbiosis caused by the oral pathogen [193].

Shao et al. reported that AD patients’ microbiota-derived extracellular vesicles increased neuroinflammation and hyperphosphorylated tau levels in the cortex and hippocampus of treated mice, as well as caused a significant cognitive decline measured in several behavioral tests in examined animals, compared to mice treated with vesicles isolated from healthy individuals or a vehicle control [194].

Microbial EVs and their contact with the host organism are a relatively novel object of scientific interest, especially in the context of their interaction with the CNS; thus, further careful examination is needed to distinguish the potential pro-health effect of probiotic EV cargo and the detrimental influence of pathogenic microbes or imbalanced microbiota.

#### 3.1.8. Other Metabolites

Methylamines are another group of bacterial metabolites which have been observed to have a beneficial effect on BBB integrity. Both in vitro and in vivo experiments have shown that methylamine trimethylamine N-oxide enhances BBB unity and shields it from harmful LPS-induced inflammation effects; however, results regarding this metabolite’s influence on neural cells are not entirely conclusive and, moreover, its processing and action may be dependent on the inflammation status [195].

**Figure 10:**
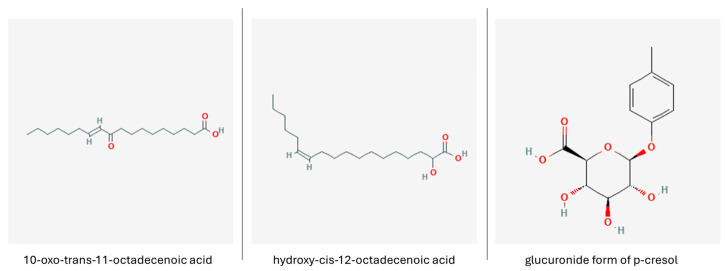
10-Oxo-11-octadecenoic acid. (PubChem 10308378 [198]). Hydroxy-*cis*-12-octadecenoic acid (PubChem 129725103 [199]). *p*-cresol glucuronide (PubChem 154035 [200]).

10-oxo-trans-11-octadecenoic acid and hydroxy-*cis*-12-octadecenoic acid (Figure 10), *L. plantarum* metabolites generated from linoleic acid, were reported to have anti-inflammatory action and decrease nitric oxide production in mouse microglia stimulated with LPS, supposedly by inhibiting ERK phosphorylation [196].

A glucuronide form of *p*-cresol (Figure 10), created from a product of bacterial fermentation of tyrosine and phenylalanine metabolized later in the liver, has been observed to protect BBB integrity under LPS assault, by acting as an antagonist of the Toll-like receptor 4 complex, in contrast to the sulfate metabolite of *p*-cresol, which is an uremic toxin with detrimental effects on the CNS [197].

Recently, it has also been proven that sirtuins, histone deacetylases influencing multiple pathways involved in the aging process and auspicious target molecules for ND therapy, are produced by LAB, as Sir2La, and *L. acidophilus* NCFM-produced sirtuin has received a detailed functional evaluation [201]. It is hypothesized that such produced proteins may be implemented in patients with sirtuin deficits [202].

Another category of bacteria metabolites gathering growing interest are quorum-sensing peptides (QSPs). QSPs, a means of bacterial communication, were also observed to selectively cross the BBB and interact with CNS cells. As they are not a homogenic group of compounds, comprising lactones, oligopeptides, furan borate derivatives and other various molecules, scientists have scant knowledge regarding the influence of QSPs on neural cells. An extensive study, encompassing cell lines of diversified origin, was carried out to assess the potential of 85 QSPs with different chemical characteristics. A total of 22 peptides exerted various effects on CNS cells; 3 QSPs were slightly cytotoxic towards neural cell lines, 6 QSPs affected PC12 cells’ neurite growth and differentiation and 3 peptides stimulated IL-6 secretion in murine microglial cells [203].

PapRIV, a *B. cereus*-derived peptide, based on in vitro experiments is implied to reach the systemic circulation and pass through the BBB. Application of the peptide to BV-2 microglial cells induced cytokine IL-6 and TNFα production, triggering a neuroinflammatory response, and elevated intracellular ROS levels. Conditioned media prepared by BV-2 treatment with PapRIV was proven to considerably decrease SH-SY5Y cell viability. Interestingly, the peptide treatment alone was not cytotoxic to either BV-2 or SH-SY5Y cells [204]. It is also believed that pathogenic bacteria-derived QSPs play a significant role in AD pathogenesis [205].

There is substantial evidence that lactic acid-producing bacteria, for instance *L. acidophilus* or *B. subtilis*, secrete QSP inhibitors, such as LAB-derived polysaccharides, bacteriocins like plantaricins or postbiotic mixture, which disable pathogenic bacterial QSP spread [206].

### 3.2. Brief Overview of Postbiotics’ Impact on Processes Involved in ND Pathogenesis

A review of the current data revealed that probiotic bacteria and their metabolites exert a multidirectional influence on cellular events involved in the pathogenesis of ND. This influence is exerted both directly, through interactions with the signaling pathways of CNS cells, and indirectly, through the modulation of their function via the “gut–brain” axis. Due to their pleotropic potential, postbiotics can be considered anti-senescent agents that protect the host organism from aging accelerators, such as oxidative stress and inflammation, as well as hamper cellular phenomena favoring senescence by inhibition of the mTOR pathway and autophagy improvement. Their actions also include reversing undesired epigenetic events facilitating senescence. Moreover, they may act as senomorphics, alleviating the results of a spreading SASP and oxi-inflammatory stress, which both promote age-related pathologies.

A postbiotic mixture and its components, which influence processes implemented in aging and neurodegeneration process, together with a brief description of their properties and exerted actions, are presented in Table 1.

## 4. Future Perspectives and Limitations

Neurodegenerative diseases, inadvertently connected with ageing, pose a great danger to modern societies, as people tend to live longer and often require special care and attention in their elderly years.

However, NDs are complex conditions that affect a variety of cells, including not only neurons, but also variegated groups of neuroglia cells and epithelial and vascular system cells, and encompass diversified processes, such as those discussed above, including oxidative stress occurrence, proteinopathy, autophagy dysfunction, cellular senescence and SASP spread, neuronal loss that cannot be sufficiently compensated, as well as a compromised BBB and intestinal barrier. Given the intricate character of ND, addressing only a part of the issue, symptomatic care, is not enough to alleviate the disease course, as a successful treatment is still to be designed. Drugs currently approved for ND therapy cannot stop or even slow the progression of these devastating disorders. Applied pharmaceuticals only relieve the symptoms of the diseases, while inducing a number of adverse effects such as gastrointestinal disorders, weight loss, increased extrapyramidal symptoms, skin allergic reactions, psychiatric disorders, cardiac arrhythmias or vascular disorders [207].

Since postbiotics comprise plenty of chemically diversified molecules that possess multiple healthful properties proven to remediate phenomena underlying ND pathogenesis, their application in ND treatment and prophylaxis should be considered. With the scientific data indicating the antioxidant, anti-inflammatory and anti-proliferative potential of postbiotics, they could also be applied as an adjunctive element of ND pharmacotherapy [208]. However, bacteria inhabiting the human organism, their interactions via, among other substances, secreted metabolites and other chemicals, together with their impact on the host’s body, are at a complexity level exceeding our current understanding; therefore, any designed postbiotic mixtures must be analyzed with great care. Although the interaction of bacterial metabolites with the host organism should be subjected to a meticulous examination, application of postbiotics is still safer than introducing viable bacteria and still well worth the effort for postbiotics’ implied neuroprotective properties. Introducing postbiotics into the combinatorial approaches for treating ND is being attempted to alleviate neuropathology by supporting traditional treatment with natural dietary ingredients and supplements [209]. Postbiotics, with many documented beneficial properties, could potentially be such adjunctive agents.

Current clinical trials regarding various components of postbiotic mixtures derived from host-friendly bacteria are mainly focused on investigating the compound’s influence on the cognitive fitness and emotional well-being of healthy individuals. Since the idea of implementing postbiotics as agents promoting CNS homeostasis is quite novel in terms of the clinical application pathway, few of the trials’ results have been published yet. So far, apart from the forementioned spermine and spermidine studies, there have been trials aimed at elucidating calcium and magnesium derivatives of butyric acid in Gulf-War Syndrome patients [210], SCFAs’ impact on the cognition and mood of healthy individuals (NCT03688854) and postbiotics’ potential to alleviate the acute stress response and improve mood and stress management, together with cognition and sleep in healthy subjects under stressful conditions [211]. Postbiotics have also been delivered to healthy adults to assess their potential in moderate anxiety management [212], while supplements containing s-equol have been applied to menopausal woman to ameliorate menopausal transition symptoms, such as working memory, attention and learning deficits and sleep quality disruption [213], as well as brain metabolism [214]. S-equol was also reported to improve mitochondrial function in AD patients [215]. A variety of health advantages of postbiotics may soon lead to an increase in consumer demand for postbiotic supplements and functional food fortified with these bioactive microbial by-products. Defining the mechanisms of postbiotic action in relation to human health may result in the future production of supplements or drugs that could be applied for preventing diseases and achieving therapeutic goals. Consequently, postbiotics seem to be a secure substitute and a novel application approach in the pharmaceutical and functional food industries; however, further research is needed to expand the knowledge in this field. A considerable body of evidence, some of which has been presented in the current article, confirms the positive influence of several probiotics and their metabolites not only on CNS function, but also on age-related phenomena, and makes postbiotics auspicious bioactive compounds to support prophylaxis and therapy for neurodegenerative disorders, which are worth translating from bench to pharmaceutical market in response to “silver consumers’” demands.

## Figures and Tables

**Table 1:** ND pathogenesis-hampering effects of postbiotic compounds on CNS cells.

	Compound	Action	References
Postculture mixture	Postculture mixture		
	Cell-free supernatant from *P. distasonis* and *M. massiliensis*	neuroprotective properties, antioxidant activity, ↑ differentiation of neural cells, ↓ pro-inflammatory cytokines, preferential protection of differentiated neurons	[116]
	*L. plantarum* fermented soy milk	↓ H_2_O_2_ cytotoxicity towards neural cells, ↑ viability, ↑ BDNF and TH, ↓ proapoptotic gene expression	[117]
	LAB-derived mixture	↓ rotenone and MPP+ cytotoxicity towards neural cells, ↑ BBB restoration, inhibition of pathogenic QSP spread	[85,118,206]
	*B. longum* derived	↑ BDNF in corticosterone-stimulated SH-SY5Y cells	[119]
Short-chain fatty acids	SCFA		
	SCFA	antioxidant and anti-inflammatory properties, modification of intestinal microbiota composition, ↑ neural cells’ maturation and function, ↓ GSK-3β activity, ↓ Aβ and tau proteinopathies, ↓ ND pathology, ↓ cognitive deficits, ↑ adult neurogenesis, ↑ BDNF, ↑ epigenetic modificaitons, ↑ glutamate-glutamine shuttle, ↑ astrocytes’ functioning, ↑ astrocyte-neuron communication, ↓ oxidative stress damage to neurons	[122,128,129,130,131,132,134]
	Acetate	histone deacetylase inhibitors (HDACis), inducing chromatin remodeling change	[122,124]
	Propionate	histone deacetylase inhibitors (HDACis) protective action toward BBB cells, ↑ BBB integrity and homeostasis	[90,123,124]
	Butyrate	histone deacetylase inhibitors (HDACis) protective action toward BBB cells, ↑ BBB integrity and homeostasis, ↑ BBB restoration	[85,90,123,124]
Lactate	Lactate		
	Lactate	neuroprotective effect, ↑ neurogenesis, but excessive accumulation ↓ neurogenesis, ↑ OPCs differentiation, ↑ histone lactylation, ↑ epigenetic modifications, metabolic pathways alterations, ↑ inflammation, ↑ NF-κB, ↑ IL-6 and IL-8, regulates the mechanism of microglia anti- or pro-inflammatory phenotype switch	[135,136,137,139,140,141,142,143,144]
Polyamines	Polyamines		
	Acetylated amines	↑ cognitive decline, ↑ tau fibrilization	[146]
	Spermidine	antioxidant, ↑ proliferation, ↑ differentiation, ↑ autophagy, ↑ neurotrophic factors, ↑ mitochondrial function, ↓ proinflammatory proteins, ↑ dementia patients’ memory performance	[147,150,154]
	Spermine	antioxidant, ↑ autophagy, ↑ neurotrophic factors, ↑ mitochondrial function, ↓ proinflammatory proteins, ↓ α-syn cytotoxicity towards dopaminergic neurons, ↓ manganese cytotoxicity	[150,151]
Trypthophan metabolites	Trypthophan metabolites		
	Indoles	↓ oxidative stress, ↓ neuroinflammation, ↓ apoptosis in neurons, ↑ neurogenesis	[157,158]
	indole acetic acid and indole propionic acid	↑ GPR30/AMPK/SIRT1 pathway, ↓ neurodegeneration process, ↑ β-catenin, ↑ Neurog2, ↑ VEGF-alpha expression	[157]
	5-hydroxyindole acetic acid and kynurenic acid	↑ neprilisin, ↑ Aβ degradation, ↓ Aβ cytotoxicity	[159]
	Nicotinamide N-oxide	↓ neuroinflammation, ↓ microglia polarization, ↑ anti-inflammatory microglia phenotype, ↑ mitophagy, ↓ glial pro-inflammatory cytokine release, ↑ sirtuin 1 expression	[160,161]
	Anthranilic acid, Hydroxykynurenine, Quinolic acid and Picolinic acid	↑ oxidative stress, ↑ neuroinflammation, ↑ neuronal apoptosis	[156]
Polyphenols	Polyphenols		
	Urolithin A	↑ BBB integrity, ↓ neuroinflammation, ↑neurogenesis, ↑ microglial phagocytosis ↑ mitophagy	[90,169,170,171]
	Dihydroxyphenyl-γ-valerolactones	↑ neuritogenesis	[171,172]
	Aryl-γ-valerolactones	↓ amyloid-β	[171,172]
	Equol	↓ microglia inflammation, ↓ LPS, 6-OHDA- and MPP+-induced cytotoxicity	[173]
Exopolysaccharides	Exopolysaccharides		
	LAB-derived	Inhibitors of pathogenic QSP	[206]
	*L. plantarum*-derived	↓ oxidative stress	[177]
	*L. delbrueckii* ssp. *Bulgaricus* B3- and *L. plantarum* GD2-derived	↑ viability, ↓ oxidative stress, ↓ Aβ1-42 cytotoxicity	[178]
	Ram12 from *L. rhamnosus*	↓ oxidative stress and pro-inflammatory markers, ↑ anti-inflammatory cytokine IL-10	[179]
Extracellular vesicles	Extracellular vesicles		
	*L. plantarum*-derived	↑ BDNF expression, ↓ stress-induced depressive behavior	[189]
	*L. rhamnosus*-derived	↓ microglia pro-inflammatory polarization, ↓ inflammatory response	[190]
	*L. paracasei*-derived	↓ Aβ-induced pathology ↑ neurotrophic factors, ↑ Aβ-degrading proteases, ↑ sirtuin 1, ↓ astrogliosis, ↑ hippocampal neurogenesis, ↓ cognitive decline	[191]
	*A. actinomycetemcomitans*	↑ pro-inflammatory cytokines, ↑ TLR4 and TLR8	[192]
	*P. gingivalis*	↑ neuroinflammation, implied in dementia pathogenesis	[193]
	AD patients’ microbiota-derived	↑ neuroinflammation, ↑ hyperphosphorylated tau, ↑ cognitive decline	[194]
Other metabolites	Other metabolites		
	methylamine trimethylamine N-oxide	↑ BBB unity, ↓ inflammation, results not conclusive	[134,195]
	10-oxo-trans-11-octadecenoic acid, hydroxy-cis-12-octadecenoic acid	anti-inflammatory action, ↓ nitric oxide production, ↓ ERK phosphorylation	[196]
	glucuronide form of *p*-cresol	↑ BBB integrity, an antagonist of Toll-like receptor 4 complex	[197]
	QSP	various effects on CNS cells including cytotoxic, varied effect on neurite growth and differentiation, ↑ IL-6, ↑ NO, ↑ AD pathogenesis	[203,205]
	PapRIV	↑ microglial cytotoxicity towards neurons	[204]

## Abbreviations

6-OHDA	6-hydroxydopamine
α-syn	synuclein α
Aβ	amyloid β
AD	Alzheimer’s disease
ATG	autophagy-related gene
BBB	blood–brain barrier
BDNF	brain-derived neurotrophic factor
BV-2	murine microglia cell line
CEBPβ	CCAA T/enhancer-binding protein β
CMA	chaperone-mediated autophagy
CNS	central nervous system
COX-2	cyclooxygenase 2
CREB	cAMP-response element-binding protein
DA	dopamine
EPS	exopolysaccharide
ERK	extracellular signal-regulated kinase
ETC	electron transport chain
EVs	extracellular vesicles
FOXO3a	Forkhead Transcription Factor O Subfamily Member 3a
GFAP	glial fibrillary acidic protein
GR	glucocorticoid receptor
GSK-3β	glycogen synthase kinase-3β
HDAC	NAD+-dependent histone deacetylase
HDACi	histone deacetylase inhibitor
HSV	*Herpes simplex* virus
IFN	interferon
IGF-1	insulin-like growth factor 1
IL	interleukin
iNOS	inducible nitric oxide synthase
LAB	lactic acid bacteria
LPS	lipopolysaccharide
LRRK2	leucine-rich repeat kinase 2
LTA	lipoteichoic acids
MAO	monoamine oxidase
MCT1	monocarboxylate transporter 1
MIP	macrophage inflammatory protein
MMP-3	metalloproteinase 3
MPP+	1-methyl-4-phenylpyridinium
mTOR	mammalian target of rapamycin
NAMO	nicotinamide N-oxide
ND	neurodegenerative disease
Neurog2	neurogenin 2
NF-κB	nuclear factor κB
NFT	neurofibrillary tangle
NO	nitric oxide
NQO1	quinone oxidoreductase 1
NRF-1	nuclear respiratory factor 1
NSC	neural stem cells
OPC	oligodendrocyte precursor cells
OS	oxidative stress
PD	Parkinson’s disease
PGC1α	peroxisome proliferator-activated receptor gamma coactivator 1-alpha
QSP	quorum sensing peptides
RNS	reactive nitrogen species
ROS	reactive oxygen species
SASP	senescence-associated secretory phenotype
SCFA	short-chain fatty acid
SGK1	serum- and glucocorticoid kinase 1
SH-SY5Y	human neuroblastoma cel line
SIRT	sirtuin
SK-MEL-28	human melanoma cell line
TFAM	mitochondrial transcription factor A
TH	tyrosine hydroxylase
TGF-β	transforming growth factor β
TLR	Toll-like receptor
TNF-α	tumor necrosis factor α
UPS	ubiquitin-proteasome system
VEGF	vascular endothelial growth factor

## References

[1] Abubakar M.B., Sanusi K.O., Ugusman A., Mohamed W., Kamal H., Ibrahim N.H., Khoo C.S., Kumar J. (2022). Alzheimer’s Disease: An Update and Insights into Pathophysiology. Front. Aging Neurosci..

[2] Kulcsarova K., Skorvanek M., Postuma R.B., Berg D. Defining Parkinson’s Disease: Past and Future. J. Park. Dis..

[3] Ahmad S.S., Waheed T., Rozeen S., Mahmood S., Kamal M.A. (2019). Therapeutic Study of Phytochemicals Against Cancer and Alzheimer’s Disease Management. Curr. Drug Metab..

[4] Baker D.J., Petersen R.C. (2018). Cellular Senescence in Brain Aging and Neurodegenerative Diseases: Evidence and Perspectives. J. Clin. Invest..

[5] Bashir B., Alam S., Khandale N., Birla D., Vishwas S., Pandey N.K., Gupta G., Paudel K.R., Dureja H., Kumar P. (2024). Opening Avenues for Treatment of Neurodegenerative Disease Using Post-Biotics: Breakthroughs and Bottlenecks in Clinical Translation. Ageing Res. Rev..

[6] Taghizadeh Ghassab F., Shamlou Mahmoudi F., Taheri Tinjani R., Emami Meibodi A., Zali M.R., Yadegar A. (2024). Probiotics and the Microbiota-Gut-Brain Axis in Neurodegeneration: Beneficial Effects and Mechanistic Insights. Life Sci..

[7] Bulacios G.A., Cataldo P.G., Naja J.R., de Chaves E.P., Taranto M.P., Minahk C.J., Hebert E.M., Saavedra M.L. (2023). Improvement of Key Molecular Events Linked to Alzheimer’s Disease Pathology Using Postbiotics. ACS Omega.

[8] Jang H.J., Lee N.-K., Paik H.-D. (2024). A Narrative Review on the Advance of Probiotics to Metabiotics. J. Microbiol. Biotechnol..

[9] Rahman M.H., Bajgai J., Fadriquela A., Sharma S., Trinh T.T., Akter R., Jeong Y.J., Goh S.H., Kim C.-S., Lee K.-J. (2021). Therapeutic Potential of Natural Products in Treating Neurodegenerative Disorders and Their Future Prospects and Challenges. Molecules.

[10] Cobley J.N., Fiorello M.L., Bailey D.M. (2018). 13 Reasons Why the Brain Is Susceptible to Oxidative Stress. Redox Biol..

[11] Rao A.V., Balachandran B. (2002). Role of Oxidative Stress and Antioxidants in Neurodegenerative Diseases. Nutr. Neurosci..

[12] Singh A., Kukreti R., Saso L., Kukreti S. (2019). Oxidative Stress: A Key Modulator in Neurodegenerative Diseases. Molecules.

[13] Butterfield D.A., Halliwell B. (2019). Oxidative Stress, Dysfunctional Glucose Metabolism and Alzheimer Disease. Nat. Rev. Neurosci..

[14] Bai R., Guo J., Ye X.-Y., Xie Y., Xie T. (2022). Oxidative Stress: The Core Pathogenesis and Mechanism of Alzheimer’s Disease. Ageing Res. Rev..

[15] Trist B.G., Hare D.J., Double K.L. (2019). Oxidative Stress in the Aging Substantia Nigra and the Etiology of Parkinson’s Disease. Aging Cell.

[16] Hemmati-Dinarvand M., Saedi S., Valilo M., Kalantary-Charvadeh A., Alizadeh Sani M., Kargar R., Safari H., Samadi N. (2019). Oxidative Stress and Parkinson’s Disease: Conflict of Oxidant-Antioxidant Systems. Neurosci. Lett..

[17] Alqahtani T., Deore S.L., Kide A.A., Shende B.A., Sharma R., Dadarao Chakole R., Nemade L.S., Kishor Kale N., Borah S., Shrikant Deokar S. (2023). Mitochondrial Dysfunction and Oxidative Stress in Alzheimer’s Disease, and Parkinson’s Disease, Huntington’s Disease and Amyotrophic Lateral Sclerosis—An Updated Review. Mitochondrion.

[18] Zhao Y., Zhao B. (2013). Oxidative Stress and the Pathogenesis of Alzheimer’s Disease. Oxid. Med. Cell Longev..

[19] Tchekalarova J., Tzoneva R. (2023). Oxidative Stress and Aging as Risk Factors for Alzheimer’s Disease and Parkinson’s Disease: The Role of the Antioxidant Melatonin. Int. J. Mol. Sci..

[20] Islam M.T. (2017). Oxidative Stress and Mitochondrial Dysfunction-Linked Neurodegenerative Disorders. Neurol. Res..

[21] Percário S., da Silva Barbosa A., Varela E.L.P., Gomes A.R.Q., Ferreira M.E.S., de Nazaré Araújo Moreira T., Dolabela M.F. (2020). Oxidative Stress in Parkinson’s Disease: Potential Benefits of Antioxidant Supplementation. Oxidative Med. Cell. Longev..

[22] Solleiro-Villavicencio H., Rivas-Arancibia S. (2018). Effect of Chronic Oxidative Stress on Neuroinflammatory Response Mediated by CD4+T Cells in Neurodegenerative Diseases. Front. Cell Neurosci..

[23] Kwon H.S., Koh S.-H. (2020). Neuroinflammation in Neurodegenerative Disorders: The Roles of Microglia and Astrocytes. Transl. Neurodegener..

[24] Kono R., Ikegaya Y., Koyama R. (2021). Phagocytic Glial Cells in Brain Homeostasis. Cells.

[25] Zhang W., Xiao D., Mao Q., Xia H. (2023). Role of Neuroinflammation in Neurodegeneration Development. Signal Transduct. Target. Ther..

[26] Gelders G., Baekelandt V., Van der Perren A. (2018). Linking Neuroinflammation and Neurodegeneration in Parkinson’s Disease. J. Immunol. Res..

[27] Çınar E., Tel B.C., Şahin G. (2022). Neuroinflammation in Parkinson’s Disease and Its Treatment Opportunities. Balk. Med. J..

[28] Wang Q., Liu Y., Zhou J. (2015). Neuroinflammation in Parkinson’s Disease and Its Potential as Therapeutic Target. Transl. Neurodegener..

[29] Reimer L., Vesterager L.B., Betzer C., Zheng J., Nielsen L.D., Kofoed R.H., Lassen L.B., Bølcho U., Paludan S.R., Fog K. (2018). Inflammation Kinase PKR Phosphorylates α-Synuclein and Causes α-Synuclein-Dependent Cell Death. Neurobiol. Dis..

[30] Kawahata I., Finkelstein D.I., Fukunaga K. (2022). Pathogenic Impact of α-Synuclein Phosphorylation and Its Kinases in α-Synucleinopathies. Int. J. Mol. Sci..

[31] Lin J., Ou R., Li C., Hou Y., Zhang L., Wei Q., Pang D., Liu K., Jiang Q., Yang T. (2023). Plasma Glial Fibrillary Acidic Protein as a Biomarker of Disease Progression in Parkinson’s Disease: A Prospective Cohort Study. BMC Med..

[32] Zhang Z., Yang X., Song Y.-Q., Tu J. (2021). Autophagy in Alzheimer’s Disease Pathogenesis: Therapeutic Potential and Future Perspectives. Ageing Res. Rev..

[33] Hou X., Watzlawik J.O., Fiesel F.C., Springer W. (2020). Autophagy in Parkinson’s Disease. J. Mol. Biol..

[34] Loeffler D.A. (2019). Influence of Normal Aging on Brain Autophagy: A Complex Scenario. Front. Aging Neurosci..

[35] Liu G., Yu Q., Tan B., Ke X., Zhang C., Li H., Zhang T., Lu Y. (2022). Gut Dysbiosis Impairs Hippocampal Plasticity and Behaviors by Remodeling Serum Metabolome. Gut Microbes.

[36] Herranz N., Gallage S., Gil J. (2015). TORn about SASP Regulation. Cell Cycle.

[37] Salotti J., Johnson P.F. (2019). Regulation of Senescence and the SASP by the Transcription Factor C/EBPβ. Exp. Gerontol..

[38] Jęśko H., Wencel P., Strosznajder R.P., Strosznajder J.B. (2017). Sirtuins and Their Roles in Brain Aging and Neurodegenerative Disorders. Neurochem. Res..

[39] Li R., Li Y., Zuo H., Pei G., Huang S., Hou Y. (2024). Alzheimer’s Amyloid-β Accelerates Cell Senescence and Suppresses SIRT1 in Human Neural Stem Cells. Biomolecules.

[40] Lilja S., Oldenburg J., Pointner A., Dewald L., Lerch M., Hippe B., Switzeny O., Haslberger A. (2020). Epigallocatechin Gallate Effectively Affects Senescence and Anti-SASP via SIRT3 in 3T3-L1 Preadipocytes in Comparison with Other Bioactive Substances. Oxid. Med. Cell Longev..

[41] Cuanalo-Contreras K., Schulz J., Mukherjee A., Park K.-W., Armijo E., Soto C. (2022). Extensive Accumulation of Misfolded Protein Aggregates during Natural Aging and Senescence. Front. Aging Neurosci..

[42] Golde T.E., Miller V.M. (2009). Proteinopathy-Induced Neuronal Senescence: A Hypothesis for Brain Failure in Alzheimer’s and Other Neurodegenerative Diseases. Alzheimers Res. Ther..

[43] Pantelis P., Theocharous G., Lagopati N., Veroutis D., Thanos D.-F., Lampoglou G.-P., Pippa N., Gatou M.-A., Tremi I., Papaspyropoulos A. (2023). The Dual Role of Oxidative-Stress-Induced Autophagy in Cellular Senescence: Comprehension and Therapeutic Approaches. Antioxidants.

[44] Schultz M.B., Sinclair D.A. (2016). When Stem Cells Grow Old: Phenotypes and Mechanisms of Stem Cell Aging. Development.

[45] Culig L., Chu X., Bohr V.A. (2022). Neurogenesis in Aging and Age-Related Neurodegenerative Diseases. Ageing Res. Rev..

[46] Hagg T. (2009). From Neurotransmitters to Neurotrophic Factors to Neurogenesis. Neuroscientist.

[47] Gould E., Reeves A.J., Graziano M.S.A., Gross C.G. (1999). Neurogenesis in the Neocortex of Adult Primates. Science.

[48] Eriksson P.S., Perfilieva E., Björk-Eriksson T., Alborn A.M., Nordborg C., Peterson D.A., Gage F.H. (1998). Neurogenesis in the Adult Human Hippocampus. Nat. Med..

[49] Salta E., Lazarov O., Fitzsimons C.P., Tanzi R., Lucassen P.J., Choi S.H. (2023). Adult Hippocampal Neurogenesis in Alzheimer’s Disease: A Roadmap to Clinical Relevance. Cell Stem Cell.

[50] Kot M., Neglur P.K., Pietraszewska A., Buzanska L. (2022). Boosting Neurogenesis in the Adult Hippocampus Using Antidepressants and Mesenchymal Stem Cells. Cells.

[51] Audesse A.J., Webb A.E. (2020). Mechanisms of Enhanced Quiescence in Neural Stem Cell Aging. Mech. Ageing Dev..

[52] Anand K.S., Dhikav V. (2012). Hippocampus in Health and Disease: An Overview. Ann. Indian. Acad. Neurol..

[53] Lim J., Bang Y., Choi H.J. (2018). Abnormal Hippocampal Neurogenesis in Parkinson’s Disease: Relevance to a New Therapeutic Target for Depression with Parkinson’s Disease. Arch. Pharm. Res..

[54] Boldrini M., Underwood M.D., Hen R., Rosoklija G.B., Dwork A.J., John Mann J., Arango V. (2009). Antidepressants Increase Neural Progenitor Cells in the Human Hippocampus. Neuropsychopharmacology.

[55] Spalding K.L., Bergmann O., Alkass K., Bernard S., Salehpour M., Huttner H.B., Boström E., Westerlund I., Vial C., Buchholz B.A. (2013). Dynamics of Hippocampal Neurogenesis in Adult Humans. Cell.

[56] Anacker C., Hen R. (2017). Adult Hippocampal Neurogenesis and Cognitive Flexibility—Linking Memory and Mood. Nat. Rev. Neurosci..

[57] Anacker C., Cattaneo A., Luoni A., Musaelyan K., Zunszain P.A., Milanesi E., Rybka J., Berry A., Cirulli F., Thuret S. (2013). Glucocorticoid-Related Molecular Signaling Pathways Regulating Hippocampal Neurogenesis. Neuropsychopharmacology.

[58] Anacker C., Cattaneo A., Musaelyan K., Zunszain P.A., Horowitz M., Molteni R., Luoni A., Calabrese F., Tansey K., Gennarelli M. (2013). Role for the Kinase SGK1 in Stress, Depression, and Glucocorticoid Effects on Hippocampal Neurogenesis. Proc. Natl. Acad. Sci. USA.

[59] Colucci-D’Amato L., Speranza L., Volpicelli F. (2020). Neurotrophic Factor BDNF, Physiological Functions and Therapeutic Potential in Depression, Neurodegeneration and Brain Cancer. Int. J. Mol. Sci..

[60] Gamit N., Dharmarajan A., Sethi G., Warrier S. (2023). Want of Wnt in Parkinson’s Disease: Could sFRP Disrupt Interplay between Nurr1 and Wnt Signaling?. Biochem. Pharmacol..

[61] Marchetti B. (2020). Nrf2/Wnt Resilience Orchestrates Rejuvenation of Glia-Neuron Dialogue in Parkinson’s Disease. Redox Biol..

[62] Arredondo S.B., Valenzuela-Bezanilla D., Mardones M.D., Varela-Nallar L. (2020). Role of Wnt Signaling in Adult Hippocampal Neurogenesis in Health and Disease. Front. Cell Dev. Biol..

[63] Marchetti B., Tirolo C., L’Episcopo F., Caniglia S., Testa N., Smith J.A., Pluchino S., Serapide M.F. (2020). Parkinson’s Disease, Aging and Adult Neurogenesis: Wnt/β-Catenin Signalling as the Key to Unlock the Mystery of Endogenous Brain Repair. Aging Cell.

[64] L’Episcopo F., Tirolo C., Serapide M.F., Caniglia S., Testa N., Leggio L., Vivarelli S., Iraci N., Pluchino S., Marchetti B. (2018). Microglia Polarization, Gene-Environment Interactions and Wnt/β-Catenin Signaling: Emerging Roles of Glia-Neuron and Glia-Stem/Neuroprogenitor Crosstalk for Dopaminergic Neurorestoration in Aged Parkinsonian Brain. Front. Aging Neurosci..

[65] Lai S., Wang P., Gong J., Zhang S. (2023). New Insights into the Role of GSK-3β in the Brain: From Neurodegenerative Disease to Tumorigenesis. PeerJ.

[66] Cope E.C., Gould E. (2019). Adult Neurogenesis, Glia, and the Extracellular Matrix. Cell Stem Cell.

[67] Andromidas F., Atashpanjeh S., Myers A.J., MacKinnon B.E., Shaffer M.M., Koob A.O. (2021). The Astrogenic Balance in the Aging Brain. Curr. Neuropharmacol..

[68] Bergles D.E., Richardson W.D. (2015). Oligodendrocyte Development and Plasticity. Cold Spring Harb. Perspect. Biol..

[69] Yeung M.S.Y., Djelloul M., Steiner E., Bernard S., Salehpour M., Possnert G., Brundin L., Frisén J. (2019). Dynamics of Oligodendrocyte Generation in Multiple Sclerosis. Nature.

[70] Grabert K., Michoel T., Karavolos M.H., Clohisey S., Baillie J.K., Stevens M.P., Freeman T.C., Summers K.M., McColl B.W. (2016). Microglial Brain Region−dependent Diversity and Selective Regional Sensitivities to Aging. Nat. Neurosci..

[71] Zhang L., Cao Y., Zhang X., Gu X., Mao Y., Peng B. (2022). The Origin and Repopulation of Microglia. Dev. Neurobiol..

[72] Marchetti B., L’Episcopo F., Morale M.C., Tirolo C., Testa N., Caniglia S., Serapide M.F., Pluchino S. (2013). Uncovering Novel Actors in Astrocyte–Neuron Crosstalk in Parkinson’s Disease: The Wnt/β-Catenin Signaling Cascade as the Common Final Pathway for Neuroprotection and Self-Repair. Eur. J. Neurosci..

[73] Jung B.-K., Park Y., Yoon B., Bae J.-S., Han S.-W., Heo J.-E., Kim D.-E., Ryu K.-Y. (2023). Reduced Secretion of LCN2 (Lipocalin 2) from Reactive Astrocytes through Autophagic and Proteasomal Regulation Alleviates Inflammatory Stress and Neuronal Damage. Autophagy.

[74] Ferreira A.C., Santos T., Sampaio-Marques B., Novais A., Mesquita S.D., Ludovico P., Bernardino L., Correia-Neves M., Sousa N., Palha J.A. (2018). Lipocalin-2 Regulates Adult Neurogenesis and Contextual Discriminative Behaviours. Mol. Psychiatry.

[75] Diaz-Aparicio I., Paris I., Sierra-Torre V., Plaza-Zabala A., Rodríguez-Iglesias N., Márquez-Ropero M., Beccari S., Huguet P., Abiega O., Alberdi E. (2020). Microglia Actively Remodel Adult Hippocampal Neurogenesis through the Phagocytosis Secretome. J. Neurosci..

[76] Nicola R., Okun E. (2021). Adult Hippocampal Neurogenesis: One Lactate to Rule Them All. Neuromol. Med..

[77] Bonfanti L., Couillard-Després S. (2023). Neuron and Brain Maturation 2.0. Int. J. Mol. Sci..

[78] Hussain G., Akram R., Anwar H., Sajid F., Iman T., Han H.S., Raza C., De Aguilar J.-L.G. (2024). Adult Neurogenesis: A Real Hope or a Delusion?. Neural Regen. Res..

[79] Moyaert P., Padrela B.E., Morgan C.A., Petr J., Versijpt J., Barkhof F., Jurkiewicz M.T., Shao X., Oyeniran O., Manson T. (2023). Imaging Blood-Brain Barrier Dysfunction: A State-of-the-Art Review from a Clinical Perspective. Front. Aging Neurosci..

[80] Alkhalifa A.E., Al-Ghraiybah N.F., Odum J., Shunnarah J.G., Austin N., Kaddoumi A. (2023). Blood–Brain Barrier Breakdown in Alzheimer’s Disease: Mechanisms and Targeted Strategies. Int. J. Mol. Sci..

[81] Kaya M., Ahishali B. (2021). Basic Physiology of the Blood-Brain Barrier in Health and Disease: A Brief Overview. Tissue Barriers.

[82] Hosseinkhani B., Duran G., Hoeks C., Hermans D., Schepers M., Baeten P., Poelmans J., Coenen B., Bekar K., Pintelon I. (2023). Cerebral Microvascular Endothelial Cell-Derived Extracellular Vesicles Regulate Blood-Brain Barrier Function. Fluids Barriers CNS.

[83] de Rus Jacquet A., Alpaugh M., Denis H.L., Tancredi J.L., Boutin M., Decaestecker J., Beauparlant C., Herrmann L., Saint-Pierre M., Parent M. (2023). The Contribution of Inflammatory Astrocytes to BBB Impairments in a Brain-Chip Model of Parkinson’s Disease. Nat. Commun..

[84] Varatharaj A., Galea I. (2017). The Blood-Brain Barrier in Systemic Inflammation. Brain Behav. Immun..

[85] Fock E., Parnova R. (2023). Mechanisms of Blood–Brain Barrier Protection by Microbiota-Derived Short-Chain Fatty Acids. Cells.

[86] Davidson T.L., Stevenson R.J. (2024). Vulnerability of the Hippocampus to Insults: Links to Blood-Brain Barrier Dysfunction. Int. J. Mol. Sci..

[87] Ivanidze J., Mackay M., Hoang A., Chi J.M., Cheng K., Aranow C., Volpe B., Diamond B., Sanelli P.C. (2019). Dynamic Contrast-Enhanced MRI Reveals Unique Blood-Brain Barrier Permeability Characteristics in the Hippocampus in the Normal Brain. AJNR Am. J. Neuroradiol..

[88] Suarez A.N., Noble E.E., Kanoski S.E. (2019). Regulation of Memory Function by Feeding-Relevant Biological Systems: Following the Breadcrumbs to the Hippocampus. Front. Mol. Neurosci..

[89] Wu Y.-C., Bogale T.A., Koistinaho J., Pizzi M., Rolova T., Bellucci A. (2024). The Contribution of β-Amyloid, Tau and α-Synuclein to Blood–Brain Barrier Damage in Neurodegenerative Disorders. Acta Neuropathol..

[90] Knox E.G., Aburto M.R., Clarke G., Cryan J.F., O’Driscoll C.M. (2022). The Blood-Brain Barrier in Aging and Neurodegeneration. Mol. Psychiatry.

[91] Wang Q., Zheng J., Pettersson S., Reynolds R., Tan E.-K. (2023). The Link between Neuroinflammation and the Neurovascular Unit in Synucleinopathies. Sci. Adv..

[92] Hotel A. Health and Nutritional Properties of Probiotics in Food Including Powder Milk with Live Lactic Acid Bacteria. Proceedings of the Joint FAO/WHO Expert Consultation.

[93] Moradi M., Molaei R., Guimarães J.T. (2021). A Review on Preparation and Chemical Analysis of Postbiotics from Lactic Acid Bacteria. Enzym. Microb. Technol..

[94] Żółkiewicz J., Marzec A., Ruszczyński M., Feleszko W. (2020). Postbiotics-A Step Beyond Pre- and Probiotics. Nutrients.

[95] Garbacz K. (2022). Anticancer Activity of Lactic Acid Bacteria. Semin. Cancer Biol..

[96] González-Lozano E., García-García J., Gálvez J., Hidalgo-García L., Rodríguez-Nogales A., Rodríguez-Cabezas M.E., Sánchez M. (2022). Novel Horizons in Postbiotics: Lactobacillaceae Extracellular Vesicles and Their Applications in Health and Disease. Nutrients.

[97] Lou X., Xue J., Shao R., Mo C., Wang F., Chen G. (2023). Postbiotics as Potential New Therapeutic Agents for Sepsis. Burn. Trauma..

[98] Descamps H.C., Herrmann B., Wiredu D., Thaiss C.A. (2019). The Path toward Using Microbial Metabolites as Therapies. eBioMedicine.

[99] Fang X., Yue M., Wei J., Wang Y., Hong D., Wang B., Zhou X., Chen T. (2021). Evaluation of the Anti-Aging Effects of a Probiotic Combination Isolated From Centenarians in a SAMP8 Mouse Model. Front. Immunol..

[100] Tsai Y.-C., Cheng L.-H., Liu Y.-W., Jeng O.-J., Lee Y.-K. (2021). Gerobiotics: Probiotics Targeting Fundamental Aging Processes. Biosci. Microbiota Food Health.

[101] Tran S.M.-S., Mohajeri M.H. (2021). The Role of Gut Bacterial Metabolites in Brain Development, Aging and Disease. Nutrients.

[102] Sharma R., Padwad Y. (2020). Probiotic Bacteria as Modulators of Cellular Senescence: Emerging Concepts and Opportunities. Gut Microbes.

[103] Rekatsina M., Paladini A., Piroli A., Zis P., Pergolizzi J.V., Varrassi G. (2020). Pathophysiology and Therapeutic Perspectives of Oxidative Stress and Neurodegenerative Diseases: A Narrative Review. Adv. Ther..

[104] Bailo P.S., Martín E.L., Calmarza P., Breva S.M., Gómez A.B., Giráldez A.P., Callau J.J.S.-P., Santamaría J.M.V., Khialani A.D., Micó C.C. (2022). The Role of Oxidative Stress in Neurodegenerative Diseases and Potential Antioxidant Therapies. Adv. Lab. Med./Av. Med. Lab..

[105] Morén C., deSouza R.M., Giraldo D.M., Uff C. (2022). Antioxidant Therapeutic Strategies in Neurodegenerative Diseases. Int. J. Mol. Sci..

[106] İncili G.K., Karatepe P., Akgöl M., Güngören A., Koluman A., İlhak O.İ., Kanmaz H., Kaya B., Hayaloğlu A.A. (2022). Characterization of Lactic Acid Bacteria Postbiotics, Evaluation in-Vitro Antibacterial Effect, Microbial and Chemical Quality on Chicken Drumsticks. Food Microbiol..

[107] Hamad G.M., Abdelmotilib N.M., Darwish A.M.G., Zeitoun A.M. (2020). Commercial Probiotic Cell-Free Supernatants for Inhibition of Clostridium Perfringens Poultry Meat Infection in Egypt. Anaerobe.

[108] Angelin J., Kavitha M. (2020). Exopolysaccharides from Probiotic Bacteria and Their Health Potential. Int. J. Biol. Macromol..

[109] Kim H.S., Chae H.S., Jeong S.G., Ham J.S., Im S.K., Ahn C.N., Lee J.M. (2005). In Vitro Antioxidative Properties of Lactobacilli. Asian Australas. Asian Australas. J. Anim. Sci..

[110] Thorakkattu P., Khanashyam A.C., Shah K., Babu K.S., Mundanat A.S., Deliephan A., Deokar G.S., Santivarangkna C., Nirmal N.P. (2022). Postbiotics: Current Trends in Food and Pharmaceutical Industry. Foods.

[111] Salvi P.S., Cowles R.A. (2021). Butyrate and the Intestinal Epithelium: Modulation of Proliferation and Inflammation in Homeostasis and Disease. Cells.

[112] Montalbán-Rodríguez A., Abalo R., López-Gómez L. (2024). From the Gut to the Brain: The Role of Enteric Glial Cells and Their Involvement in the Pathogenesis of Parkinson’s Disease. Int. J. Mol. Sci..

[113] Kocot A.M., Jarocka-Cyrta E., Drabińska N. (2022). Overview of the Importance of Biotics in Gut Barrier Integrity. Int. J. Mol. Sci..

[114] Askarova S., Umbayev B., Masoud A.-R., Kaiyrlykyzy A., Safarova Y., Tsoy A., Olzhayev F., Kushugulova A. (2020). The Links Between the Gut Microbiome, Aging, Modern Lifestyle and Alzheimer’s Disease. Front. Cell Infect. Microbiol..

[115] Kim H., Kim S., Park S., Park G., Shin H., Park M.S., Kim J. (2021). Administration of Bifidobacterium Bifidum BGN4 and Bifidobacterium Longum BORI Improves Cognitive and Memory Function in the Mouse Model of Alzheimer’s Disease. Front. Aging Neurosci..

[116] Ahmed S., Busetti A., Fotiadou P., Vincy Jose N., Reid S., Georgieva M., Brown S., Dunbar H., Beurket-Ascencio G., Delday M.I. (2019). In Vitro Characterization of Gut Microbiota-Derived Bacterial Strains With Neuroprotective Properties. Front. Cell. Neurosci..

[117] Choi G.-H., Bock H.-J., Lee N.-K., Paik H.-D. (2022). Soy Yogurt Using Lactobacillus Plantarum 200655 and Fructooligosaccharides: Neuroprotective Effects against Oxidative Stress. J. Food Sci. Technol..

[118] Michael D.R., Davies T.S., Loxley K.E., Allen M.D., Good M.A., Hughes T.R., Plummer S.F. (2019). In Vitro Neuroprotective Activities of Two Distinct Probiotic Consortia. Benef. Microbes.

[119] Han S.-K., Kim D.H. (2019). Lactobacillus Mucosae and Bifidobacterium Longum Synergistically Alleviate Immobilization Stress-Induced Anxiety/Depression in Mice by Suppressing Gut Dysbiosis. J. Microbiol. Biotechnol..

[120] Hor Y.-Y., Ooi C.-H., Khoo B.-Y., Choi S.-B., Seeni A., Shamsuddin S., Oon C.-E., Ong K.-L., Jeong W.-S., Liong M.-T. (2019). Lactobacillus Strains Alleviated Aging Symptoms and Aging-Induced Metabolic Disorders in Aged Rats. J. Med. Food.

[121] Chakraborty P., Gamage H.K.A.H., Laird A.S. (2024). Butyrate as a Potential Therapeutic Agent for Neurodegenerative Disorders. Neurochem. Int..

[122] Dugan B., Conway J., Duggal N.A. (2023). Inflammaging as a Target for Healthy Ageing. Age Ageing.

[123] Licciardi P.V., Ververis K., Karagiannis T.C. (2011). Histone Deacetylase Inhibition and Dietary Short-Chain Fatty Acids. Int. Sch. Res. Not..

[124] Yuille S., Reichardt N., Panda S., Dunbar H., Mulder I.E. (2018). Human Gut Bacteria as Potent Class I Histone Deacetylase Inhibitors in Vitro through Production of Butyric Acid and Valeric Acid. PLoS ONE.

[125] National Center for Biotechnology Information PubChem Compound Summary for CID 176, Acetic Acid. https://pubchem.ncbi.nlm.nih.gov/compound/Acetic-Acid.

[126] National Center for Biotechnology Information PubChem Compound Summary for CID 1032, Propionic Acid. https://pubchem.ncbi.nlm.nih.gov/compound/Propionic-Acid.

[127] National Center for Biotechnology Information PubChem Compound Summary for CID 264, Butyric Acid. https://pubchem.ncbi.nlm.nih.gov/compound/Butyric-Acid.

[128] Flynn C.M., Yuan Q. (2023). Probiotic Supplement as a Promising Strategy in Early Tau Pathology Prevention: Focusing on GSK-3β?. Front. Neurosci..

[129] Song X., Zhao Z., Zhao Y., Wang Z., Wang C., Yang G., Li S. (2022). Lactobacillus Plantarum DP189 Prevents Cognitive Dysfunction in D-Galactose/AlCl_3_ Induced Mouse Model of Alzheimer’s Disease via Modulating Gut Microbiota and PI3K/Akt/GSK-3β Signaling Pathway. Nutr. Neurosci..

[130] Zhou Y., Xie L., Schröder J., Schuster I.S., Nakai M., Sun G., Sun Y.B.Y., Mariño E., Degli-Esposti M.A., Marques F.Z. (2023). Dietary Fiber and Microbiota Metabolite Receptors Enhance Cognition and Alleviate Disease in the 5xFAD Mouse Model of Alzheimer’s Disease. J. Neurosci..

[131] Bull-Larsen S., Mohajeri M.H. (2019). The Potential Influence of the Bacterial Microbiome on the Development and Progression of ADHD. Nutrients.

[132] Feitelson M.A., Arzumanyan A., Medhat A., Spector I. (2023). Short-Chain Fatty Acids in Cancer Pathogenesis. Cancer Metastasis Rev..

[133] Gao Y., Xie D., Wang Y., Niu L., Jiang H. (2022). Short-Chain Fatty Acids Reduce Oligodendrocyte Precursor Cells Loss by Inhibiting the Activation of Astrocytes via the SGK1/IL-6 Signalling Pathway. Neurochem. Res..

[134] Sun Y., Zhang H., Zhang X., Wang W., Chen Y., Cai Z., Wang Q., Wang J., Shi Y. (2023). Promotion of Astrocyte-Neuron Glutamate-Glutamine Shuttle by SCFA Contributes to the Alleviation of Alzheimer’s Disease. Redox Biol..

[135] Lev-Vachnish Y., Cadury S., Rotter-Maskowitz A., Feldman N., Roichman A., Illouz T., Varvak A., Nicola R., Madar R., Okun E. (2019). L-Lactate Promotes Adult Hippocampal Neurogenesis. Front. Neurosci..

[136] Zhou J., Liu T., Guo H., Cui H., Li P., Feng D., Hu E., Huang Q., Yang A., Zhou J. (2018). Lactate Potentiates Angiogenesis and Neurogenesis in Experimental Intracerebral Hemorrhage. Exp. Mol. Med..

[137] Ichihara Y., Doi T., Ryu Y., Nagao M., Sawada Y., Ogata T. (2017). Oligodendrocyte Progenitor Cells Directly Utilize Lactate for Promoting Cell Cycling and Differentiation. J. Cell Physiol..

[138] National Center for Biotechnology Information PubChem Compound Summary for CID 612, Lactic Acid. https://pubchem.ncbi.nlm.nih.gov/compound/Lactic-Acid.

[139] Scandella V., Knobloch M. (2019). Sensing the Environment: Extracellular Lactate Levels Control Adult Neurogenesis. Cell Stem Cell.

[140] Zhang D., Tang Z., Huang H., Zhou G., Cui C., Weng Y., Liu W., Kim S., Lee S., Perez-Neut M. (2019). Metabolic Regulation of Gene Expression by Histone Lactylation. Nature.

[141] Yu X., Yang J., Xu J., Pan H., Wang W., Yu X., Shi S. (2024). Histone Lactylation: From Tumor Lactate Metabolism to Epigenetic Regulation. Int. J. Biol. Sci..

[142] Pan R.-Y., He L., Zhang J., Liu X., Liao Y., Gao J., Liao Y., Yan Y., Li Q., Zhou X. (2022). Positive Feedback Regulation of Microglial Glucose Metabolism by Histone H4 Lysine 12 Lactylation in Alzheimer’s Disease. Cell Metab..

[143] Wei L., Yang X., Wang J., Wang Z., Wang Q., Ding Y., Yu A. (2023). H3K18 Lactylation of Senescent Microglia Potentiates Brain Aging and Alzheimer’s Disease through the NFκB Signaling Pathway. J. Neuroinflamm..

[144] Han H., Zhao Y., Du J., Wang S., Yang X., Li W., Song J., Zhang S., Zhang Z., Tan Y. (2023). Exercise Improves Cognitive Dysfunction and Neuroinflammation in Mice through Histone H3 Lactylation in Microglia. Immun. Ageing.

[145] Ji X., Yu W., Jin M., Lu L., Yin H., Wang H. (2024). Possible Role of Cellular Polyamine Metabolism in Neuronal Apoptosis. Curr. Med. Sci..

[146] Sandusky-Beltran L.A., Kovalenko A., Ma C., Calahatian J.I.T., Placides D.S., Watler M.D., Hunt J.B., Darling A.L., Baker J.D., Blair L.J. (2019). Spermidine/Spermine-N1-Acetyltransferase Ablation Impacts Tauopathy-Induced Polyamine Stress Response. Alzheimers Res. Ther..

[147] Hirano R., Shirasawa H., Kurihara S. (2021). Health-Promoting Effects of Dietary Polyamines. Med. Sci..

[148] Noack J., Dongowski G., Hartmann L., Blaut M. (2000). The Human Gut Bacteria Bacteroides Thetaiotaomicron and Fusobacterium Varium Produce Putrescine and Spermidine in Cecum of Pectin-Fed Gnotobiotic Rats. J. Nutr..

[149] Arena M.E., Manca de Nadra M.C. (2001). Biogenic Amine Production by Lactobacillus. J. Appl. Microbiol..

[150] Xu T.-T., Li H., Dai Z., Lau G.K., Li B.-Y., Zhu W.-L., Liu X.-Q., Liu H.-F., Cai W.-W., Huang S.-Q. (2020). Spermidine and Spermine Delay Brain Aging by Inducing Autophagy in SAMP8 Mice. Aging.

[151] Vijayan B., Raj V., Nandakumar S., Kishore A., Thekkuveettil A. (2019). Spermine Protects Alpha-Synuclein Expressing Dopaminergic Neurons from Manganese-Induced Degeneration. Cell Biol. Toxicol..

[152] Schwarz C., Benson G.S., Horn N., Wurdack K., Grittner U., Schilling R., Märschenz S., Köbe T., Hofer S.J., Magnes C. (2022). Effects of Spermidine Supplementation on Cognition and Biomarkers in Older Adults With Subjective Cognitive Decline: A Randomized Clinical Trial. JAMA Netw. Open.

[153] Pekar T., Bruckner K., Pauschenwein-Frantsich S., Gschaider A., Oppliger M., Willesberger J., Ungersbäck P., Wendzel A., Kremer A., Flak W. (2021). The Positive Effect of Spermidine in Older Adults Suffering from Dementia. Wien. Klin. Wochenschr..

[154] Pekar T., Wendzel A., Jarisch R. (2024). The Positive Effect of Spermidine in Older Adults Suffering from Dementia after 1 Year. Wien. Klin. Wochenschr..

[155] Gao J., Xu K., Liu H., Liu G., Bai M., Peng C., Li T., Yin Y. (2018). Impact of the Gut Microbiota on Intestinal Immunity Mediated by Tryptophan Metabolism. Front. Cell. Infect. Microbiol..

[156] Shaw C., Hess M., Weimer B.C. (2023). Microbial-Derived Tryptophan Metabolites and Their Role in Neurological Disease: Anthranilic Acid and Anthranilic Acid Derivatives. Microorganisms.

[157] Yin J., Zhang Y., Liu X., Li W., Hu Y., Zhang B., Wang S. (2023). Gut Microbiota-Derived Indole Derivatives Alleviate Neurodegeneration in Aging through Activating GPR30/AMPK/SIRT1 Pathway. Mol. Nutr. Food Res..

[158] Wei G.Z., Martin K.A., Xing P.Y., Agrawal R., Whiley L., Wood T.K., Hejndorf S., Ng Y.Z., Low J.Z.Y., Rossant J. (2021). Tryptophan-Metabolizing Gut Microbes Regulate Adult Neurogenesis via the Aryl Hydrocarbon Receptor. Proc. Natl. Acad. Sci. USA.

[159] Maitre M., Klein C., Patte-Mensah C., Mensah-Nyagan A.-G. (2020). Tryptophan Metabolites Modify Brain Aβ Peptide Degradation: A Role in Alzheimer’s Disease?. Prog. Neurobiol..

[160] Li F., Wang Y., Song X., Wang Z., Jia J., Qing S., Huang L., Wang Y., Wang S., Ren Z. (2022). The Intestinal Microbial Metabolite Nicotinamide N-Oxide Prevents Herpes Simplex Encephalitis via Activating Mitophagy in Microglia. Gut Microbes.

[161] Song X., Cao W., Wang Z., Li F., Xiao J., Zeng Q., Wang Y., Li S., Ye C., Wang Y. (2022). Nicotinamide N-Oxide Attenuates HSV-1-Induced Microglial Inflammation through Sirtuin-1/NF-κB Signaling. Int. J. Mol. Sci..

[162] Schwarcz R., Foo A., Sathyasaikumar K.V., Notarangelo F.M. (2024). The Probiotic Lactobacillus Reuteri Preferentially Synthesizes Kynurenic Acid from Kynurenine. Int. J. Mol. Sci..

[163] National Center for Biotechnology Information PubChem Compound Summary for CID 3744, 3-Indolepropionic acid. https://pubchem.ncbi.nlm.nih.gov/compound/3-Indolepropionic-acid.

[164] National Center for Biotechnology Information PubChem Compound Summary for CID 8617, Indole-3-butyric acid. https://pubchem.ncbi.nlm.nih.gov/compound/Indole-3-butyric-acid.

[165] National Center for Biotechnology Information PubChem Compound Summary for CID 3845, Kynurenic acid. https://pubchem.ncbi.nlm.nih.gov/compound/Kynurenic-acid.

[166] National Center for Biotechnology Information PubChem Compound Summary for CID 72661, Nicotinamide N-oxide. https://pubchem.ncbi.nlm.nih.gov/compound/Nicotinamide-N-oxide.

[167] Láng L., McArthur S., Lazar A.S., Pourtau L., Gaudout D., Pontifex M.G., Müller M., Vauzour D. (2024). Dietary (Poly)Phenols and the Gut–Brain Axis in Ageing. Nutrients.

[168] Sharma R. (2022). Emerging Interrelationship Between the Gut Microbiome and Cellular Senescence in the Context of Aging and Disease: Perspectives and Therapeutic Opportunities. Probiotics Antimicro. Prot..

[169] Lin X.-H., Ye X.-J., Li Q.-F., Gong Z., Cao X., Li J.-H., Zhao S.-T., Sun X.-D., He X.-S., Xuan A.-G. (2020). Urolithin A Prevents Focal Cerebral Ischemic Injury via Attenuating Apoptosis and Neuroinflammation in Mice. Neuroscience.

[170] González-Sarrías A., Núñez-Sánchez M.Á., Tomás-Barberán F.A., Espín J.C. (2017). Neuroprotective Effects of Bioavailable Polyphenol-Derived Metabolites against Oxidative Stress-Induced Cytotoxicity in Human Neuroblastoma SH-SY5Y Cells. J. Agric. Food Chem..

[171] Leyrolle Q., Prado-Perez L., Layé S. (2023). The Gut-Derived Metabolites as Mediators of the Effect of Healthy Nutrition on the Brain. Front. Nutr..

[172] Ruotolo R., Minato I., La Vitola P., Artioli L., Curti C., Franceschi V., Brindani N., Amidani D., Colombo L., Salmona M. (2020). Flavonoid-Derived Human Phenyl-γ-Valerolactone Metabolites Selectively Detoxify Amyloid-β Oligomers and Prevent Memory Impairment in a Mouse Model of Alzheimer’s Disease. Mol. Nutr. Food Res..

[173] Johnson S.L., Park H.Y., Vattem D.A., Grammas P., Ma H., Seeram N.P. (2020). Equol, a Blood-Brain Barrier Permeable Gut Microbial Metabolite of Dietary Isoflavone Daidzein, Exhibits Neuroprotective Effects against Neurotoxins Induced Toxicity in Human Neuroblastoma SH-SY5Y Cells and Caenorhabditis Elegans. Plant Foods Hum. Nutr..

[174] National Center for Biotechnology Information PubChem Compound Summary for CID 5488186, Urolithin A. https://pubchem.ncbi.nlm.nih.gov/compound/urolithin-A.

[175] National Center for Biotechnology Information PubChem Compound Summary for CID 91469, Equol. https://pubchem.ncbi.nlm.nih.gov/compound/Equol.

[176] National Center for Biotechnology Information PubChem Substance Record for SID 433987232, Lactobacillus Rhamnosus GG Exopolysaccharide Hexasaccharide Repeating Unit, Source: BioCyc. https://pubchem.ncbi.nlm.nih.gov/substance/433987232..

[177] Sirin S., Aslim B. (2021). Protective Effect of Exopolysaccharides from Lactic Acid Bacteria against Amyloid Beta1-42induced Oxidative Stress in SH-SY5Y Cells: Involvement of the AKT, MAPK, and NF-κB Signaling Pathway. Process Biochem..

[178] Li J.-Y., Jin M.-M., Meng J., Gao S.-M., Lu R.-R. (2013). Exopolysaccharide from Lactobacillus Planterum LP6: Antioxidation and the Effect on Oxidative Stress. Carbohydr. Polym..

[179] Kumari M., Dasriya V.L., Nataraj B.H., Nagpal R., Behare P.V. (2022). Lacticaseibacillus Rhamnosus-Derived Exopolysaccharide Attenuates D-Galactose-Induced Oxidative Stress and Inflammatory Brain Injury and Modulates Gut Microbiota in a Mouse Model. Microorganisms.

[180] Singh H., Chopra C., Singh H., Malgotra V., Khurshid Wani A., Singh Dhanjal D., Sharma I., Nepovimova E., Alomar S., Singh R. (2024). Gut-Brain Axis and Alzheimer’s Disease: Therapeutic Interventions and Strategies. J. Funct. Foods.

[181] Chronopoulos A., Kalluri R. (2020). Emerging Role of Bacterial Extracellular Vesicles in Cancer. Oncogene.

[182] Liu H., Zhang Q., Wang S., Weng W., Jing Y., Su J. (2021). Bacterial Extracellular Vesicles as Bioactive Nanocarriers for Drug Delivery: Advances and Perspectives. Bioact. Mater..

[183] Xie J., Haesebrouck F., Van Hoecke L., Vandenbroucke R.E. (2023). Bacterial Extracellular Vesicles: An Emerging Avenue to Tackle Diseases. Trends Microbiol..

[184] Modasia A.A., Jones E.J., Martel L.M.-P., Louvel H., Couraud P.-O., Blackshaw L.A., Carding S.R. (2023). The Use of a Multicellular in Vitro Model to Investigate Uptake and Migration of Bacterial Extracellular Vesicles Derived from the Human Gut Commensal Bacteroides Thetaiotaomicron. J. Extracell. Biol..

[185] Li M., Lee K., Hsu M., Nau G., Mylonakis E., Ramratnam B. (2017). Lactobacillus-Derived Extracellular Vesicles Enhance Host Immune Responses against Vancomycin-Resistant Enterococci. BMC Microbiol..

[186] Zhang Z., Liu Z., Lv A., Fan C. (2023). How Toll-like Receptors Influence Parkinson’s Disease in the Microbiome–Gut–Brain Axis. Front. Immunol..

[187] Heidari A., Yazdanpanah N., Rezaei N. (2022). The Role of Toll-like Receptors and Neuroinflammation in Parkinson’s Disease. J. Neuroinflamm..

[188] Frederiksen H.R., Haukedal H., Freude K. (2019). Cell Type Specific Expression of Toll-Like Receptors in Human Brains and Implications in Alzheimer’s Disease. BioMed Res. Int..

[189] Choi J., Kim Y.-K., Han P.-L. (2019). Extracellular Vesicles Derived from Lactobacillus Plantarum Increase BDNF Expression in Cultured Hippocampal Neurons and Produce Antidepressant-like Effects in Mice. Exp. Neurobiol..

[190] Yang Y., Li N., Gao Y., Xu F., Chen H., Zhang C., Ni X. (2024). The Activation Impact of Lactobacillus-Derived Extracellular Vesicles on Lipopolysaccharide-Induced Microglial Cell. BMC Microbiol..

[191] Kwon H., Lee E.-H., Park S.-Y., Park J.-Y., Hong J.-H., Kim E.-K., Shin T.-S., Kim Y.-K., Han P.-L. (2023). Lactobacillus-Derived Extracellular Vesicles Counteract Aβ42-Induced Abnormal Transcriptional Changes through the Upregulation of MeCP2 and Sirt1 and Improve Aβ Pathology in Tg-APP/PS1 Mice. Exp. Mol. Med..

[192] Ha J.Y., Seok J., Kim S.-J., Jung H.-J., Ryu K.-Y., Nakamura M., Jang I.-S., Hong S.-H., Lee Y., Lee H.-J. (2023). Periodontitis Promotes Bacterial Extracellular Vesicle-Induced Neuroinflammation in the Brain and Trigeminal Ganglion. PLoS Pathog..

[193] Ma X., Yoo J.-W., Shin Y.-J., Park H.-S., Son Y.-H., Kim D.-H. (2023). Alleviation of Porphyromonas Gingivalis or Its Extracellular Vesicles Provoked Periodontitis and Cognitive Impairment by Lactobacillus Pentosus NK357 and Bifidobacterium Bifidum NK391. Nutrients.

[194] Shao Z., Lu Y., Xing A., He X., Xie H., Hu M. (2024). Effect of Outer Membrane Vesicles of Lactobacillus Pentosus on Tau Phosphorylation and CDK5-Calpain Pathway in Mice. Exp. Gerontol..

[195] Wen X., Dong H., Zou W. (2024). The Role of Gut Microorganisms and Metabolites in Intracerebral Hemorrhagic Stroke: A Comprehensive Review. Front. Neurosci..

[196] Ikeguchi S., Izumi Y., Kitamura N., Kishino S., Ogawa J., Akaike A., Kume T. (2018). Inhibitory Effect of the Gut Microbial Linoleic Acid Metabolites, 10-Oxo-Trans-11-Octadecenoic Acid and 10-Hydroxy-Cis-12-Octadecenoic Acid, on BV-2 Microglial Cell Activation. J. Pharmacol. Sci..

[197] Stachulski A.V., Knausenberger T.B.-A., Shah S.N., Hoyles L., McArthur S. (2023). A Host–Gut Microbial Amino Acid Co-Metabolite, *p*-Cresol Glucuronide, Promotes Blood–Brain Barrier Integrity in Vivo. Tissue Barriers.

[198] National Center for Biotechnology Information PubChem Compound Summary for CID 10308378, 10-Oxo-11-octadecenoic Acid. https://pubchem.ncbi.nlm.nih.gov/compound/10-Oxo-11-octadecenoic-acid.

[199] National Center for Biotechnology Information PubChem Compound Summary for CID 129725103, Hydroxy-cis-12-octadecenoic Acid. https://pubchem.ncbi.nlm.nih.gov/compound/Hydroxy-cis-12-octadecenoic-acid.

[200] National Center for Biotechnology Information PubChem Compound Summary for CID 154035, p-Cresol Glucuronide. https://pubchem.ncbi.nlm.nih.gov/compound/p-Cresol-glucuronide.

[201] Olesen S.V., Rajabi N., Svensson B., Olsen C.A., Madsen A.S. (2018). An NAD+-Dependent Sirtuin Depropionylase and Deacetylase (Sir2La) from the Probiotic Bacterium Lactobacillus Acidophilus NCFM. Biochemistry.

[202] Sharma R. (2021). Bioactive Food Components for Managing Cellular Senescence in Aging and Disease: A Critical Appraisal and Perspectives. PharmaNutrition.

[203] Janssens Y., Wynendaele E., Verbeke F., Debunne N., Gevaert B., Audenaert K., Van DeWiele C., De Spiegeleer B. (2018). Screening of Quorum Sensing Peptides for Biological Effects in Neuronal Cells. Peptides.

[204] Janssens Y., Debunne N., De Spiegeleer A., Wynendaele E., Planas M., Feliu L., Quarta A., Claes C., Van Dam D., De Deyn P.P. (2021). PapRIV, a BV-2 Microglial Cell Activating Quorum Sensing Peptide. Sci. Rep..

[205] Dicks L.M.T. (2022). How Does Quorum Sensing of Intestinal Bacteria Affect Our Health and Mental Status?. Microorganisms.

[206] Salman M.K., Abuqwider J., Mauriello G. (2023). Anti-Quorum Sensing Activity of Probiotics: The Mechanism and Role in Food and Gut Health. Microorganisms.

[207] Walczak-Nowicka Ł.J., Herbet M. (2021). Acetylcholinesterase Inhibitors in the Treatment of Neurodegenerative Diseases and the Role of Acetylcholinesterase in Their Pathogenesis. Int. J. Mol. Sci..

[208] Liang B., Xing D. (2023). The Current and Future Perspectives of Postbiotics. Probiotics Antimicro. Prot..

[209] Andrade S., Ramalho M.J., Loureiro J.A., Pereira M. (2019). do C. Natural Compounds for Alzheimer’s Disease Therapy: A Systematic Review of Preclinical and Clinical Studies. Int. J. Mol. Sci..

[210] Microbiome Targeted Oral Butyrate Therapy in Gulf War Multisymptom Illness, (NCT05367245), Posted 10 May 2022. NCT05367245.

[211] Dalile B., Vervliet B., Bergonzelli G., Verbeke K., Van Oudenhove L. (2020). Colon-delivered short-chain fatty acids attenuate the cortisol response to psychosocial stress in healthy men: A randomized, placebo-controlled trial. Neuropsychopharmacology.

[212] A Randomized, Double-blinded, Placebo-controlled, Parallel Pilot Study, to Assess the Effect of a Postbiotic Blend on Symptoms of Anxiety in Healthy Adults With Self-Reported Mild to Moderate Anxiety, (NCT05562739), Posted 3 October 2022. NCT05562739.

[213] PhytoSERM for Menopausal Hot Flashes and Sustained Brain Health: A Double-Blind, Randomized, Placebo-Controlled Phase 2 Clinical Trial, (NCT06186531), posted 2 January 2024. NCT06186531.

[214] PhytoSERM Efficacy to Prevent Menopause Associated Decline in Brain Metabolism and Cognition: A Double-Blind, Randomized, Placebo-Controlled Phase 2 Clinical Trial, (NCT05664477), Posted 23 December 2022. NCT05664477.

[215] Wilkins H.M., Mahnken J.D., Welch P., Bothwell R., Koppel S., Jackson R.L., Burns J.M., Swerdlow R.H. (2017). A Mitochondrial Biomarker-Based Study of S-Equol in Alzheimer’s Disease Subjects: Results of a Single-Arm, Pilot Trial. J. Alzheimer’s Dis..

